# Supplementary material for: Cortical and Subcortical Neuroanatomical Signatures of Schizotypy in 3,004 Individuals Assessed in a Worldwide ENIGMA Study
Source: Mol Psychiatry. Author manuscript; Available in PMC 2022 May 2. (PMC9054674; doi:10.1038/s41380-021-01359-9)
Supplement: Supplemental Material [file EMS136389-supplement-Supplemental_Material.pdf]

Cortical and Subcortical Neuroanatomical Signatures of Schizotypy in 3,004 Individuals Assessed in a Worldwide ENIGMA Study

Kirschner and Hodzic-Santor et al.

Supplement

Index

Supplementary Figures

- Figure S1: Location of Enigma Schizotypy Working Group Members

Supplementary Methods

Site Information and Demographics

- Table S1: Demographics Cortical Meta-Analysis
- Table S2: Demographics Subcortical Meta-Analysis
- Table S3: Schizotypy Scales
- Table S4: Mean Cortical Thickness and Surface Area
- Table S5: Mean Subcortical Volume
- Table S6: Scanner Details

Meta-analyses

- Table S7: Cortical Thickness Continuous Model - No Thickness Covariate
- Table S8: Cortical Surface Area Continuous Model - No Surface Area Covariate
- Table S9: Effect of Schizotypy in Subgroup with Smoking Data
- Table S10: Effect of Schizotypy Controlling for Smoking Status
- Table S11: Effect of Schizotypy in Subgroup with Smoking Data
- Table S12: Effect of Schizotypy Controlling for Smoking Status
- Table S13: Schizotypy Questionaire Moderator
- Table S14: Scanner Field Strength Moderator
- Table S15: Scanner Number Moderator
- Table S16: FreeSurfer Version Moderator
- Table S17: Cortical Thickness Continuous Model
- Table S18: Cortical Surface Area Continuous Model
- Table S19: Subcortical Correlation Model

Funding and Acknowledgments

Supplementary Figures

Figure S1: Location of Enigma Schizotypy Working Group Members

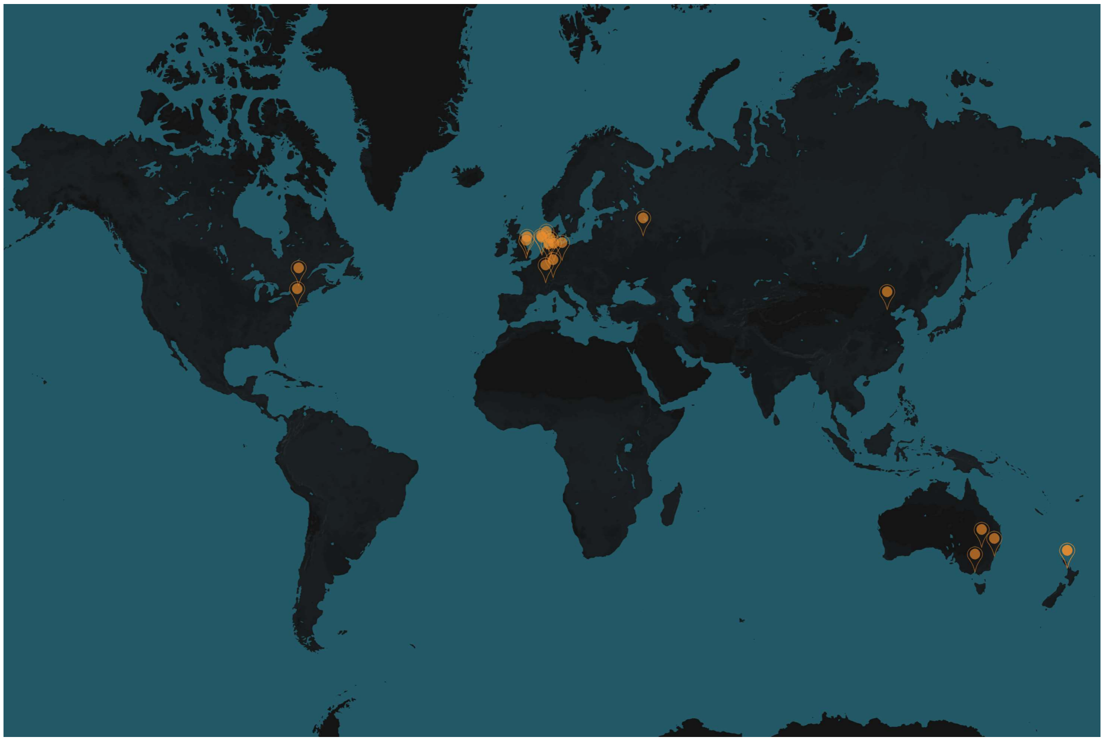

Location of Enigma Schizotypy Working Group Members

Supplementary Methods

Site Demographics & Information

Table S1: Demographics Cortical Meta-Analysis

Site	N	Scale	Female/Male	Mean Age	Age S.D.	Age Range	Mean SPT score	Score S.D.	Score range
AMS	49	CAPE	23/26	21.36	3.08	16.18-32.24	64.33	10.19	46-86
ASRB	191	SPQ	91/100	39.69	13.77	18-65	10.24	9.11	0-43
AUK	49	OLIFE	32/17	23.33	4.5	18-38	42.71	17.85	10-81
BEI	146	SPQ	77/69	19.57	1.08	16-24	23.19	12.67	2-53
BONNOLIFE	31	OLIFE	13/18	23.61	4.52	19-43	11.48	8.04	1-24
BONNRISC	51	RISC	18/33	27.2	5.36	19-40	22.37	7.71	2-34
BONNSPQ	92	SPQ	46/46	28.16	8.01	19-50	8.4	7.19	0-35
CAM	89	SPQ	48/41	24.07	5.3	1-41	16.17	10.13	0-43
DECOP	26	CAPE	09/17	38.62	7.34	28-54	66.73	12.78	50-101
FOR2107-MR	446	SPQB	279/167	34.67	12.85	18-65	3.49	3.02	0-16
FOR2107-MS	228	SPQB	146/82	28.42	10.41	18-65	3.04	2.96	0-16
GENEVA	118	SPQ	58/60	17.02	4.22	12.06-33.76	19.6	12.54	1-57
GRON1	42	SPQ	16/26	22.67	2.26	18-27	33.64	5.22	25-44
GRON2	83	CAPE-Adj	47/36	32.43	9.53	19-55	0.96	1.29	0-4.12
IGPS	63	SPQ	28/35	36.43	11.05	20.15-59.03	12.21	11.12	0-46

Site	N	Scale	Female/Male	Mean Age	Age S.D.	Age Range	Mean SPT score	Score S.D.	Score range
JENA	115	SPQ	60/55	29.37	9.65	20-60	10.76	8.21	0-49
LOND1a	46	OLIFE	22/24	27.46	6.84	18-44	26.52	15.48	4-58
LOND1b	40	CAPE	19/21	20.8	3.76	18-39	1.44	0.34	1-2.15
LOND5	44	SPQ	32/12	20.09	1.7	18-27	26.55	20.54	0-63
LOND3	40	SPQ	16/24	30.97	10.87	19-51	7.86	5.12	0-19
MELB	392	CAPE	224/168	23.38	5.17	18-50	25.43	4	20-38
MTL	40	CHAP	27/13	19.82	1.14	18.1-22.8	24.73	14.41	4-55
MOS	33	SPQ	16/17	23.83	3.35	18.1-30.1	15	11.84	1-45
MNC	173	SPQB	92/81	43.31	11.85	18-64	3.34	3.08	0-21
NYC	225	CAPE-Adj	110/115	35.92	12.85	18.47-68.04	1.14	0.16	1-1.84
UTR	97	SPQ	62/35	41.26	14.26	18-65	17.62	14.29	0-57
ZUR1	27	SPQ	10/17	29.15	10.35	19-48	42.56	7.06	34-60
ZUR2	62	SPQ	0/62	27.74	4.74	20-38	18.5	10.9	2-46
Overall	**3038**		**560.01/247.25**	**29.89**	**4.3**	**12.06-67.78**			

Table S2: Demographics Subcortical Meta-Analysis

Site	N	Scale	Female/Male	Mean Age	Age S.D.	Age Range	Mean SPT score	Score S.D.	Score range
AMS	49	CAPE	23/26	21.36	3.08	16.18-32.24	64.33	10.19	46-86
ASRB	191	SPQ	100/91	39.69	13.77	18-65	10.24	9.11	0-43
AUK	49	OLIFE	32/17	23.33	4.5	18-38	42.71	17.85	10-81
BEI	146	SPQ	77/69	19.57	1.08	16-24	23.19	12.67	2-53
BONNOLIFE	31	OLIFE	13/18	23.61	4.52	19-43	11.48	8.04	1-24
BONNRISC	55	RISC	18/37	26.73	5.5	18-40	22.35	7.59	2-34
BONNSPQ	118	SPQ	58/60	27.18	7.92	18-50	8.29	7.04	0-35
CAM	89	SPQ	48/41	24.29	4.7	18-41	16.17	10.13	0-43
DECOP	26	CAPE	9/17	38.62	7.34	28-54	66.73	12.78	50-101
FOR2107-MR	446	SPQB	279/167	34.67	12.85	18-65	3.49	3.02	0-16
FOR2107-MS	228	SPQB	146/82	28.42	10.41	18-65	3.04	2.96	0-16
GENEVA	118	SPQ	58/60	17.02	4.22	12.06-33.76	19.6	12.54	1-57
GRON1	42	SPQ	16/26	22.67	2.26	18-27	33.64	5.22	25-44
GRON2	38	CAPE-Adj	19/19	36.61	9.42	20-53	1.74	1.29	0-4.05
IGPS	63	SPQ	28/35	36.43	11.05	20.15-59.03	12.21	11.12	0-46
JENA	115	SPQ	60/55	29.37	9.65	20-60	10.76	8.21	0-49

Site	N	Scale	Female/Male	Mean Age	Age S.D.	Age Range	Mean SPT score	Score S.D.	Score range
LOND1a	46	OLIFE	22/24	27.46	6.84	18-44	26.52	15.48	4-58
LOND1b	40	CAPE	19/21	20.8	3.76	18-39	1.44	0.34	1-2.15
LOND3	40	SPQ	16/24	30.97	10.87	19-51	7.86	5.12	0-19
LOND5	30	SPQ	22/8	19.73	1.31	18-23	27.03	20.55	0-57
MELB	387	CAPE	221/166	23.38	5.15	18-50	25.38	3.92	20-38
MTL	40	CHAP	27/13	19.92	1.21	18-23	24.73	14.41	4-55
MOS	33	SPQ	16/17	23.83	3.35	18.1-30.1	15	11.84	1-45
MNC	173	SPQB	92/81	43.31	11.85	18-64	3.34	3.08	0-21
NYC	225	CAPE-Adj	110/115	35.92	12.85	18.47-68.04	1.14	0.16	1-1.84
UTR	97	SPQ	62/35	41.26	14.26	18-65	17.62	14.29	0-57
ZUR1	27	SPQ	10/17	29.15	10.35	19-48	42.56	7.06	34-60
ZUR2	62	SPQ	0/62	27.74	4.74	20-38	18.5	10.9	2-46
Overall	**3004**		**561.09/246.91**	**29.9**	**4.31**	**12.06-67.78**			

Table S3: Schizotypy Scales

Questionnaires	Total number of items	Factors/Subscales
SPQ	74	Cognitive/Perceptual; Interpersonal; Disorganized
SPQ-B	22	Cognitive/Perceptual; Interpersonal; Disorganized
CAPE	42	Depression, Negative, Positive Symptoms
OLIFE	104	Unusual Experiences; Cognitive Disorganization; Introvertive Anhedonia; Impulsive Nonconformity
RISC	26	Positive and Cognitive content of schizotypy
CHAP	196	Perceptual Aberration; Magical Ideation; Physical Anhedonia; Social Anhedonia; Impulsive Nonconformity

[a] Note. CHAP=Chapman scales; CAPE=Community Assessment of Psychotic Experiences;
Schizotypal Personality Questionnaire=SPQ; OLIFE=Oxford-Liverpool Inventory of Feelings and Experiences;
RISC=Rust Inventory of Schizotypal Cognitions.

Table S4: Mean Cortical Thickness and Surface Area

	Thickness					Surface				
Region	N	Mean	S.D.	Min	Max	N	Mean	S.D.	Min	Max
L_bankssts	2788	2.54	0.09	1.93	2.66	2824	1053.87	50.74	495	1299
L_caudalanteriorcingulate	2943	2.75	0.12	1.97	2.71	2979	662.19	31.27	312	744
L_caudalmiddlefrontal	2945	2.63	0.08	2	2.68	2981	2362.07	90.72	1252	2957
L_cuneus	2815	1.89	0.09	1.28	1.75	2851	1534.11	79.68	700	1734

	Thickness					Surface				
Region	N	Mean	S.D.	Min	Max	N	Mean	S.D.	Min	Max
L_entorhinal	2781	3.3	0.19	1.87	3.46	2816	443.49	36.78	207	586
L_fusiform	2911	2.74	0.13	1.97	2.67	2947	3301.29	179.12	1811	3997
L_inferiorparietal	2856	2.51	0.08	1.91	2.4	2892	4694.9	186.41	2377	5829
L_inferiortemporal	2882	2.82	0.17	2	2.65	2918	3480.87	243.74	1581	4170
L_isthmuscingulate	2957	2.53	0.11	1.79	2.55	2993	1028.75	38.47	587	1258
L_lateraloccipital	2915	2.22	0.11	1.5	1.95	2951	5041	319.83	3310	5992
L_lateralorbitofrontal	2931	2.7	0.09	2.1	2.81	2967	2737.12	119.92	1446	3118
L_lingual	2894	2.04	0.09	1.41	1.92	2930	3141.08	163.91	1571	3487
L_medialorbitofrontal	2915	2.48	0.11	1.86	2.45	2951	1896.48	107.96	1064	2248
L_middletemporal	2780	2.93	0.14	2.11	2.94	2816	3229.17	195.65	1725	3904
L_parahippocampal	2942	2.83	0.12	1.62	2.95	2977	708.66	36.88	432	828
L_paracentral	2954	2.45	0.1	1.81	2.26	2990	1370.63	54.69	806	1586
L_parsopercularis	2931	2.67	0.09	1.63	2.65	2967	1694.64	59.98	1032	1970
L_parsorbitalis	2935	2.8	0.11	1.89	2.71	2971	677.84	49.49	396	781
L_parstriangularis	2920	2.54	0.09	1.33	2.57	2956	1342.39	64.32	691	1718
L_pericalcarine	2868	1.63	0.11	1.09	1.58	2904	1428.78	77.26	776	1726
L_postcentral	2889	2.13	0.06	1.72	2.03	2925	4229.85	161.74	2762	4920
L_posteriorcingulate	2956	2.59	0.1	1.92	2.52	2990	1209.35	41.41	741	1469
L_precentral	2910	2.63	0.1	1.95	2.57	2945	4913.82	215.13	3315	5822
L_precuneus	2942	2.44	0.08	1.88	2.37	2978	3881.81	139.88	2551	4795
L_rostralanteriorcingulate	2929	2.94	0.12	1.96	2.94	2965	862.72	30.94	344	1129
L_rostralmiddlefrontal	2936	2.44	0.1	1.89	2.41	2972	5902.13	294.36	3692	6981
L_superiorfrontal	2936	2.82	0.1	2.14	2.67	2971	7446.57	313.1	4887	8644
L_superiorparietal	2895	2.24	0.07	1.65	2.18	2931	5524.96	194.19	3462	6325
L_superiortemporal	2767	2.87	0.11	2.25	3.04	2804	3920.64	182.28	2326	4589
L_supramarginal	2802	2.62	0.09	1.98	2.59	2838	4056.47	227.54	2192	4949
L_frontalpole	2962	2.85	0.14	1.66	3.01	2998	233.95	28.24	110	266
L_temporalpole	2911	3.58	0.22	1.77	3.8	2946	481.69	21.75	253	577
L_transversetemporal	2962	2.43	0.13	1.37	2.37	2998	466.68	21.39	273	587
L_insula	2889	3.1	0.11	2.31	2.99	2925	2328.64	139.5	1462	2466
R_bankssts	2893	2.66	0.1	1.71	2.71	2929	957.45	54.15	542	1132
R_caudalanteriorcingulate	2954	2.6	0.11	1.81	2.71	2989	770.34	40.67	329	978
R_caudalmiddlefrontal	2938	2.59	0.09	1.96	2.68	2974	2205.54	111.71	1188	2674

	Thickness					Surface				
Region	N	Mean	S.D.	Min	Max	N	Mean	S.D.	Min	Max
R_cuneus	2840	1.92	0.09	1.3	1.85	2876	1586.95	82.89	560	1931
R_entorhinal	2702	3.43	0.19	2.1	3.5	2736	388	32.51	114	477
R_fusiform	2918	2.76	0.15	1.82	2.71	2954	3227.61	166.49	1883	3809
R_inferiorparietal	2854	2.55	0.09	1.95	2.56	2891	5557.38	241.94	3172	6593
R_inferiortemporal	2892	2.84	0.18	1.65	2.79	2928	3325.58	221.94	1412	4042
R_isthmuscingulate	2946	2.51	0.09	1.86	2.6	2981	937.19	29.27	530	1146
R_lateraloccipital	2909	2.28	0.12	1.52	2.13	2945	4946.39	333.68	2909	5814
R_lateralorbitofrontal	2907	2.66	0.1	1.94	2.58	2943	2701.92	138.52	1519	3259
R_lingual	2895	2.08	0.09	1.55	2.01	2931	3194.39	165.49	1816	3708
R_medialorbitofrontal	2895	2.47	0.12	1.83	2.49	2930	1916.35	100.58	1090	2242
R_middletemporal	2864	2.96	0.13	2.06	2.95	2902	3560.95	195.57	2133	4085
R_parahippocampal	2944	2.79	0.12	1.91	2.92	2980	685.3	41.3	341	797
R_paracentral	2951	2.47	0.11	1.85	2.29	2987	1545.79	63.41	987	1723
R_parsopercularis	2908	2.66	0.08	2.03	2.64	2944	1423.51	55.76	829	1690
R_parsorbitalis	2933	2.77	0.1	1.65	2.79	2969	828.11	47.05	510	1008
R_parstriangularis	2899	2.52	0.09	1.85	2.5	2935	1544.28	75.87	895	1800
R_pericalcarine	2850	1.63	0.11	1.12	1.56	2886	1564.36	80.51	692	1855
R_postcentral	2904	2.1	0.06	1.69	2.14	2941	4068.26	152.31	2493	4747
R_posteriorcingulate	2957	2.56	0.09	1.95	2.59	2991	1232.3	46.22	702	1457
R_precentral	2908	2.58	0.09	1.94	2.54	2944	4950.88	209.46	3359	5707
R_precuneus	2941	2.45	0.08	1.88	2.42	2977	4059.22	153.04	2584	4856
R_rostralanteriorcingulate	2904	2.91	0.12	1.94	3.05	2940	672.22	49.95	273	797
R_rostralmiddlefrontal	2914	2.37	0.11	1.88	2.26	2950	6094.3	304.84	3918	7580
R_superiorfrontal	2935	2.78	0.1	2.13	2.73	2971	7218.26	305.46	4387	7915
R_superiorparietal	2911	2.23	0.07	1.71	2.17	2947	5501.62	204.91	3317	6671
R_superiortemporal	2842	2.9	0.1	2.19	2.96	2878	3705.84	162.91	2382	4175
R_supramarginal	2829	2.63	0.09	1.98	2.57	2865	3770.22	144.57	2139	4604
R_frontalpole	2953	2.8	0.14	1.57	2.95	2988	301.61	21.78	140	367
R_temporalpole	2848	3.67	0.24	1.75	4	2884	442.52	27.47	204	538
R_transversetemporal	2965	2.46	0.15	1.42	2.46	3001	350.23	16.54	167	425
R_insula	2856	3.08	0.13	2.31	3.03	2892	2331.06	106.23	1449	2653
LThickness	2967	2.54	0.08	2.08	2.36	3003	12.71	48.22	2.08	2.36
RThickness	2967	2.54	0.08	2.04	2.4	3003	13.31	46.87	2.04	2.4

	Thickness					Surface				
**Region**	**N**	**Mean**	**S.D.**	**Min**	**Max**	**N**	**Mean**	**S.D.**	**Min**	**Max**
**LSurfArea**	2967	87267.07	3148.57	59365.8	97806.3	3003	87333.3	3265.94	59365.8	97806.3
**RSurfArea**	2967	87544.61	3106.6	59252	96576.2	3003	87608.73	3224.42	59252	96576.2

Table S5: Mean Subcortical Volume

Region	N	Mean	S.D.	Min	Max
**LLatVent**	2997	7456.26	1053.33	1369.30	10534.10
**RLatVent**	2997	6978.64	1023.95	1264.90	12906.20
**Lthal**	2973	7953.10	482.56	4685.70	8519.10
**Lcaud**	2988	3761.75	197.85	2036.90	4058.80
**Lput**	2941	5478.50	458.59	2175.20	4843.50
**Lpal**	2769	1748.65	299.01	419.10	982.60
**Lhippo**	2977	4291.68	238.80	2098.30	4625.80
**Lamyg**	2979	1673.98	100.81	1044.40	1778.70
**Laccumb**	2972	592.75	169.58	141.20	409.60
**Rthal**	2993	7357.46	572.20	2729.20	4381.60
**Rcaud**	2989	3796.49	269.10	1447.00	2487.30
**Rput**	2972	5309.39	564.15	1125.50	1922.70
**Rpal**	2977	1814.57	670.70	723.00	2003.20
**Rhippo**	2985	4407.02	226.90	2484.00	4867.30
**Ramyg**	2967	1789.44	284.99	1074.00	2024.30
**Raccumb**	2970	610.73	65.95	260.70	637.50
**Mvent**	2996	7212.76	1027.45	1471.70	11361.40
**Mthal**	2970	7655.74	459.63	4568.80	6598.60
**Mcaud**	2981	3777.99	141.06	2185.80	4088.90
**Mput**	2927	5391.41	485.77	1994.60	3327.75
**Mpal**	2764	1792.46	350.06	833.05	1930.80
**Mhippo**	2973	4349.02	217.64	2627.85	4689.60
**Mamyg**	2957	1730.82	175.00	1113.55	1908.60
**Maccumb**	2951	601.66	103.30	231.25	507.95
**ICV**	2999	1533873.22	68257.29	769523.00	1710000.00

Table S6: Scanner Details

Sample Abbreviation	Sample Name	Number of Scanners	Scanner Type	Imaging protocols	Slice Orientation	FreeSurfer Version	Operating System
AMS	Amsterdam	1	3T Phillips Achieva	3D T1-weighted images (TR = 8.2, TE = 3.8, FA = 8°, FOV 240 × 188 mm, voxel size 1 × 1 × 1, 220 slices)	sequential ascending	6.0.1	Virtual linux environmen (singularity on HPC)
ASRB	Sydney 1	5	1.5T Siemens Avanto	3D MPRAGE; TR 1980ms, TE 4.3ms, field-of-view 250 x 250 mm2, data acquisition matrix 256 x 256, 176 contiguous 1mm slices; voxel size 0.98 x 0.98 x 1.0 mm3, flip angle 15°	sagittal	5.1	Mac OS X 10.9.5
AUCK	Aukland	1	3T Siemens	3D MPRAGE; FOV = 256 mm2; matrix size = 256x 256 mm; number of slices = 176; slice thickness = 1 mm; voxel-size = 1 x1 x1 mm3; TE/TR/flip angle = 2.07 ms/1900 ms/9deg; GRAPPA acceleration factor = 2; TA = 4.26 min.	interleaved	5.3.0	Linux
BEIJ	Beijing	2	3T Siemens	site 1: MPRAGE; TR 2530ms, TE 2.34ms, FOV 256mm, matrix 256x256, 192 slices, slice thickness 1mm, flip angle 7; site 2: MPRAGE; TR 2530ms, TE 2.34ms, FOV 256mm, matrix 256x256, 172 slices, slice thickness 1mm, flip angle 7;	sagittal	6.0.0	Linux
BONNOLIFE	Bonn O-Life	1	3T Siemens Trio	TR = 1570 ms; TE = 3.42 ms; inversion time (TI) = 800 ms; flip angle = 15°; FoV = 256 mm; matrix size = 256 x 256; 160 slices; slice thickness = 1 mm; voxel size = 1 x 1 x 1	sagittal	6.0.0	Virtual linux environmen (singularity on HPC)
BONNRISC	Bonn RISC	2	1.5 T GE Signa Advantage, 3T Siemens Magnetom Verio	GE Signa Advantage: 3D-SPGR voxel resolution: 1 x 1 x 1.5 mm; repetition time (TR): 18 ms; inversion time (TI): 450 ms; echo time (TE): 5.1 ms; bandwidth: 15.63 kHz	sagittal	6.0.0	Virtual linux environmen (singularity on HPC)
BONNSPQ	Bonn SPQ	1	3T Siemens Magnetom Verio	3D MPRAGE sequence, repetition time of TR = 2400 ms, echo time TE = 3.06 ms, flip angle = 9 degrees with 160 slices, slice thickness = 1.0 mm, voxel size = 1.0 × 1.0 × 1.0 mm, field of view FOV = 256 mm	sagittal	6.0.0	Virtual linux environmen (singularity on HPC)
CAM	Cambridge	1	3T Siemens Trio	MPRAGE; TR/TE 2.98/2300 ms, 1 × 1 voxels, slice thickness 1 mm, flip angle 9°, FOV 24 × 25.6 mm, 176 slices	saggital	6.0.0	Unix
DECOP	London 4	1	3T GE	T1-weighted image (196 slices; isotropic voxels of 1.2 mm; TR 7.312 ms; TE 3.016 ms;flip angle: 11°; FOV 270 mm)	sagittal	5.3	Red Hat Enterprise Linux Serve release 5.1 (Tikanga)
FOR2107-MR	Marburg	1	3T Siemens Magnetom TiroTim syngo	3D T1-weighted magnetization prepared rapid acquisition gradient echo (MPRAGE); TR=1900ms, TE=2.26ms, TI=900ms, FA=9°, voxel size=1.0x1.0x1.0mm³, Acquisition Direction Sagittal, 176 slices, slice gap 0.5mm.	sagittal	5.3	Red Hat Enterprise Linux Serve release 5.1 (Tikanga)

Sample Abbreviation	Sample Name	Number of Scanners	Scanner Type	Imaging protocols	Slice Orientation	FreeSurfer Version	Operating System
FOR2107-MS	Muenster	1	3T Siemens PRISMA	Site Muenster: 3D T1-weighted magnetization prepared rapid acquisition gradient echo (MPRAGE); TR=2130ms, TE=2.28ms, TI=900ms, FA=8°, voxel size=1.0x1.0x1.0mm³, Acquisition Direction Sagittal, 192 slices, no slice gap.	descending (anterior to posterior)	6.0.0	Mac OS SIERRA v 10.12.6
GENEVA	Geneva	2	3T Siemens Trio	T1-Weighted images (192 slices, TR=2500ms, TE=3ms, flip angle=(degree, slice hickness=1.1mm, FOV=22cm, Acq matrix 256x256)	descending	6.0.1	Virtual linux environmen (singularity on HPC)
GRON1	Groningen 1	1	3T Phillips	a T1-weighted image was obtained (TR/TE?=?9/3.5?ms) using fast-field echo and turbo-field echo: 170 axial slices; FOV (rl, ap, fh)?=?232?×?170?×?256 mm; flip angle?=?8°, voxel size?=?1?×?1?×?1 mm, slice thickness?=?1 mm.	descending	6.0.1	Virtual linux environmen (singularity on HPC)
GRON2	Groningen 2	1	3T Phillips	anatomic images were obtained using a sagittal 3-dimensional T1-weighted sequence (176 slices, repetition time 9 ms, echo time 3.5 ms, field of view 256 mm, voxel size 1 × 1 × 1 mm, slice thickness 1.0 mm) using an 8 channel head coil.	sagittal	5.3	Mac OS X 10.9.5
IGP	Sydney 2	1	3T Phillips Achieva TX	3D MPRAGE; TR 8.9ms, TE 4.1ms, field of view 240mm, matrix 268 x 268, 200 slices, slice thickness 0.9mm, no gap	sagittal	6.0.0	Linux
JENA1	Jena	1	3T Siemens Tim Trio	3D T1-weighted MPRAGE, TR: 2300ms, TE: 3.03 ms, flip angle: 9°, 192 slices, field of view 256mm, voxel resolution 1x1x1mm, acquisition time: 5:21min	sequential (top down)	6.0.0	Unix
LOND1a	London 1a	1	3T GE Discovery MR750	3D T1-weighted inversion recovery prepared gradient echo sequence (voxel size: 1.05 × 1.05 × 1.2 mm, field of view: 270 mm, 196 slices, TR: 7.3 ms, TE: 3.0 ms, inversion time: 400 ms, flip angle = 11°), based on the well-validated ADNI 2/ADNI GO protocols (see http://adni.loni.usc.edu/methods/documents/mri-protocols/ (http://adni.loni.usc.edu/methods/documents/mri-protocols/)).	interleaved	6.0.0	Unix
LOND1b	London 1b	1	3T Phillips Intera	T1-weighted 3D fast-field echo (FFE) sequence [repetition time (TR)=25 ms, echo time (TE)=4.6 ms, field of view (FOV)=260 mm, matrix=256x256, 160 contiguous axial slices of 1-mm thickness, voxel size= 1x1x1 mm].	axial	6.0.0	Unix
LOND2	London 2	2	1.5 T GE, 3T GE	1.5 T GE: 3D, TR=11.1 ms, TE=4.9 ms, inversion time=300 ms, acquisition matrix=256 x 160, 150 locations, slice thickness=1.1 mm, in-plane resolution=1.094 mm, flip angle=18°; 3 T GE: TR: 7.3 ms, TE: 3.0 ms, inversion time: 400 ms, voxel size: 1.05 x1.05 x1.2mm, field of view: 270mm, 196 slices, flip angle = 11°	interleaved	6.0.0	Mac

Sample Abbreviation	Sample Name	Number of Scanners	Scanner Type	Imaging protocols	Slice Orientation	FreeSurfer Version	Operating System
LOND5	London 5	1	3T Siemens	MPRAGE; TR 1900ms; 1mm x 1mm 1mm voxel size; in plane resolution of 256 x 256 x 176 slices, flip angle 11, slice thickness 1mm, TE = 2.07ms	sequential (top down)	6.0.0	Unix
LONDRoe	London 3	1	3T Siemens	MPRAGE; TR 2000ms; 1mm x 1mm 1mm. 256 x 256 x 176 slices, flip angle 11, slice thickness 1mm, TE = 2.07ms	interleaved	6.0.0	Linux Ubun Bionic
MELB	Melbourne	1	3T Siemens	3D magnetic-prepared rapid gradient echo sequence. A total of 192 slices were acquired for each participant's T1-weighted images using an ascending acquisition with the following parameters: TR of 2300 ms, TE of 2.07 ms, flip angle of 9°, FOV of 256mm, and voxel size of 1mm3.	sagittal	5.3.0	Linux
MNC	Muenster Neuroimaging Cohort	1	3T scanner Gyroscan Intera, Philips Medical Systems	3D fast gradient echo sequence (turbo field echo), TR = 7.4 ms, TE = 3.4 ms, Flip Angle = 9°, two signal averages, inversion prepulse every 814.5 ms, acquired over a FOV of 256 (feet-head [FH]) × 204 (anterior-posterior [AP]) × 160 (right-left [RL]) mm, phase encoding in AP and RL direction, reconstructed to cubic voxels of .5 × .5 × .5 mm	sagittal	5.3	Red Hat Enterprise Linux Serve release 5.1 (Tikanga)
MOSC	Moscow	1	3 T Phillips Ingenia	3D T1-weighted TFE; TR=7.9 ms, TE=3.5 ms; flip angle 8; number of slices=170; voxel size=0.98 x 0.98 x 1.0, no gap	axial	5.3	LinuxCent(6.10
MTL	Montreal	1	3T Siemens Trio	3D T1-weighted images were acquired with a gradient echo T1-weighted sequence, voxel size 1x1x1 mm3. TR=1900ms; TE=4.9ms; FA=25; matrix 176x256x256	saggital	6.0.0	Unix
NYC	New York	1	3T GE	3D-SPGR images using a 1mm thick slice acquisition with image parameters: TR = 7.5 ms, TE = 3 ms, matrix = 256x256, FOV = 240 mm, 216 contiguous images.	interleaved	6.0.0	Linux
UTR	Utrecht	1	3T Phillips Achieva	T1-weighted images were acquired with a 3D T1 turbo field-echo sequence. The acquisition protocol parameters were: 160 slices; repetition time = 9.96 ms; echo time = 4.59 ms; flip angle 8°; 1 mm slice thickness with no inter-slice gap; matrix = 256 × 256 and field of view 224 mm, achieving a voxel size of 0.875 × 0.875 × 1 mm. Scan time was 8 min and 50 s	saggital	5.1.0	Unix
ZUR1	Zurich 1	1	3T Phillips	3D T1-weighted images were acquired with an ultrafast gradient echo T1-weighted sequence (TR=8.4ms, TE=3.8ms, flip angle=8°) in 160 sagittal plan slices (1mm slice thickness, no slice gap) of 240×240mm2 resulting in 1x1x1mm3 voxels.	sagittal	6.0.0	Unix
ZUR2	Zurich 2	1	3T Phillips Achieva	3D T1-weighted images; 160 slices; TR, 8.2 ms; TE, 3.8 ms; flip angle, 8°; spatial resolution, 1 × 1 × 1 mm3; FOV = 160 × 240 mm2	sagittal	6.0.0	OS X

Meta-analyses

Note - Measures of heterogeneity

Tau^2: Represents the absolute value of the true variance of the effect sizes, between-study variance

The I^2 statistic: Represents the proportion (in percentage) of total variation in the estimates that can be attributed to heterogeneity among the effects rather than sampling variability. The I^2 may be interpreted according to the following ranges I^2 = 0-40%: low heterogeneity, I^2 = 30-60%: moderate heterogeneity, I^2 = 50-90%: substantial heterogeneity, I^2 = 75-100%: considerable heterogeneity

The H^2 statistic: Represents the ratio of the total amount of variability in the effect size estimates to the amount of sampling variability

Table S7: Cortical Thickness Continuous Model - No Thickness Covariate

Region	N	Effect Size (r)	SE	Lower CI	Upper CI	tau^2	I^2	H^2	pvalue	FDRp
L_bankssts	2746	0.016	0.029	-0.040	0.073	0.010	48.214	1.931	0.567	0.969
L_caudalanteriorcingulate	2884	0.017	0.019	-0.019	0.054	0.000	0.000	1.000	0.356	0.969
L_caudalmiddlefrontal	2886	-0.004	0.031	-0.064	0.057	0.013	57.288	2.341	0.907	0.969
L_cuneus	2777	-0.004	0.026	-0.055	0.047	0.006	36.154	1.566	0.878	0.969
L_entorhinal	2755	-0.022	0.019	-0.059	0.016	0.000	0.000	1.000	0.257	0.969
L_fusiform	2870	-0.028	0.023	-0.073	0.018	0.004	26.235	1.356	0.237	0.969
L_inferiorparietal	2828	-0.012	0.019	-0.049	0.025	0.000	0.000	1.000	0.524	0.969
L_inferiortemporal	2843	-0.010	0.019	-0.047	0.026	0.000	0.000	1.000	0.577	0.969
L_isthmuscingulate	2898	-0.022	0.027	-0.075	0.032	0.008	45.032	1.819	0.422	0.969
L_lateraloccipital	2866	0.030	0.025	-0.019	0.079	0.005	35.385	1.548	0.236	0.969
L_lateralorbitofrontal	2873	0.004	0.027	-0.048	0.056	0.007	41.370	1.706	0.878	0.969
L_lingual	2842	-0.018	0.019	-0.055	0.020	0.000	2.970	1.031	0.357	0.969
L_medialorbitofrontal	2862	0.051	0.022	0.008	0.095	0.002	19.860	1.248	0.021	0.246
L_middletemporal	2756	-0.013	0.019	-0.050	0.024	0.000	0.000	1.000	0.493	0.969
L_parahippocampal	2888	0.012	0.028	-0.043	0.067	0.009	48.631	1.947	0.669	0.969
L_paracentral	2897	-0.040	0.021	-0.082	0.001	0.002	15.386	1.182	0.057	0.514
L_parsopercularis	2875	-0.005	0.024	-0.052	0.042	0.004	30.249	1.434	0.838	0.969
L_parsorbitalis	2885	0.003	0.019	-0.033	0.040	0.000	0.000	1.000	0.868	0.969
L_parstriangularis	2873	-0.014	0.034	-0.080	0.052	0.018	64.460	2.814	0.685	0.969
L_pericalcarine	2811	-0.020	0.025	-0.068	0.029	0.005	30.743	1.444	0.426	0.969
L_postcentral	2838	-0.017	0.027	-0.070	0.036	0.008	43.400	1.767	0.521	0.969
L_posteriorcingulate	2896	0.023	0.019	-0.013	0.060	0.000	0.000	1.000	0.208	0.969
L_precentral	2855	-0.022	0.022	-0.064	0.020	0.002	16.485	1.197	0.307	0.969
L_precuneus	2887	-0.003	0.023	-0.047	0.042	0.003	22.911	1.297	0.899	0.969
L_rostralanteriorcingulate	2875	0.036	0.019	-0.001	0.072	0.000	0.000	1.000	0.056	0.514
L_rostralmiddlefrontal	2886	-0.012	0.024	-0.059	0.035	0.004	29.607	1.421	0.620	0.969
L_superiorfrontal	2879	-0.010	0.019	-0.046	0.027	0.000	0.000	1.000	0.597	0.969

Region	N	Effect Size (r)	SE	Lower CI	Upper CI	tau^2	I^2	H^2	pvalue	FDRp
L_superiorparietal	2851	-0.023	0.020	-0.063	0.016	0.001	8.610	1.094	0.252	0.969
L_superiortemporal	2733	-0.002	0.028	-0.056	0.052	0.008	43.695	1.776	0.945	0.991
L_supramarginal	2775	-0.027	0.019	-0.064	0.010	0.000	0.000	1.000	0.149	0.969
L_frontalpole	2902	0.044	0.018	0.008	0.080	0.000	0.000	1.000	0.017	0.233
L_temporalpole	2864	0.020	0.019	-0.017	0.056	0.000	0.000	1.000	0.290	0.969
L_transversetemporal	2903	0.035	0.021	-0.007	0.077	0.002	16.767	1.201	0.101	0.779
L_insula	2829	0.000	0.025	-0.048	0.049	0.005	32.748	1.487	0.997	0.997
R_bankssts	2840	0.026	0.027	-0.026	0.079	0.008	43.210	1.761	0.327	0.969
R_caudalanteriorcingulate	2897	0.024	0.029	-0.033	0.081	0.011	51.777	2.074	0.412	0.969
R_caudalmiddlefrontal	2884	-0.002	0.022	-0.046	0.042	0.003	21.706	1.277	0.927	0.981
R_cuneus	2793	-0.012	0.024	-0.059	0.035	0.004	28.547	1.400	0.618	0.969
R_entorhinal	2695	0.003	0.021	-0.038	0.044	0.001	9.055	1.100	0.893	0.969
R_fusiform	2877	0.000	0.024	-0.046	0.046	0.004	27.405	1.378	0.991	0.997
R_inferiorparietal	2828	0.008	0.028	-0.047	0.062	0.008	45.382	1.831	0.784	0.969
R_inferiortemporal	2855	-0.020	0.029	-0.077	0.037	0.010	51.045	2.043	0.495	0.969
R_isthmuscingulate	2890	-0.047	0.019	-0.084	-0.011	0.000	0.000	1.000	0.011	0.164
R_lateraloccipital	2868	0.006	0.028	-0.048	0.060	0.008	45.453	1.833	0.830	0.969
R_lateralorbitofrontal	2853	0.007	0.032	-0.056	0.069	0.014	58.990	2.438	0.835	0.969
R_lingual	2843	-0.023	0.027	-0.075	0.029	0.007	41.524	1.710	0.386	0.969
R_medialorbitofrontal	2847	0.061	0.019	0.025	0.097	0.000	0.000	1.000	0.001	0.037
R_middletemporal	2832	0.023	0.029	-0.033	0.078	0.010	48.635	1.947	0.430	0.969
R_parahippocampal	2897	-0.005	0.024	-0.052	0.043	0.005	32.269	1.476	0.848	0.969
R_paracentral	2894	-0.019	0.029	-0.076	0.038	0.010	51.473	2.061	0.515	0.969
R_parsopercularis	2859	0.004	0.019	-0.032	0.041	0.000	0.000	1.000	0.813	0.969
R_parsorbitalis	2882	-0.010	0.030	-0.068	0.049	0.012	53.737	2.162	0.745	0.969
R_parstriangularis	2853	0.001	0.019	-0.036	0.038	0.000	0.000	1.000	0.958	0.995
R_pericalcarine	2795	0.019	0.032	-0.044	0.082	0.015	59.607	2.476	0.558	0.969
R_postcentral	2852	-0.024	0.024	-0.071	0.024	0.004	29.856	1.426	0.326	0.969
R_posteriorcingulate	2900	0.013	0.018	-0.024	0.049	0.000	0.000	1.000	0.493	0.969
R_precentral	2859	-0.041	0.028	-0.096	0.015	0.010	49.213	1.969	0.153	0.969
R_precuneus	2886	-0.014	0.031	-0.074	0.046	0.013	56.638	2.306	0.653	0.969
R_rostralanteriorcingulate	2853	0.030	0.024	-0.017	0.076	0.004	27.177	1.373	0.209	0.969
R_rostralmiddlefrontal	2867	-0.002	0.019	-0.039	0.034	0.000	0.000	1.000	0.903	0.969
R_superiorfrontal	2881	-0.001	0.024	-0.047	0.045	0.004	27.735	1.384	0.974	0.997

Region	N	Effect Size (r)	SE	Lower CI	Upper CI	tau^2	I^2	H^2	pvalue	FDRp
R_superiorparietal	2866	-0.016	0.028	-0.071	0.039	0.009	48.389	1.938	0.571	0.969
R_superiortemporal	2802	0.011	0.023	-0.034	0.056	0.003	22.902	1.297	0.644	0.969
R_supramarginal	2794	0.000	0.019	-0.037	0.037	0.000	0.000	1.000	0.990	0.997
R_frontalpole	2894	0.049	0.018	0.012	0.085	0.000	0.000	1.000	0.008	0.152
R_temporalpole	2820	0.017	0.024	-0.029	0.063	0.004	25.975	1.351	0.466	0.969
R_transversetemporal	2905	0.011	0.023	-0.035	0.057	0.004	27.639	1.382	0.637	0.969
R_insula	2797	-0.014	0.026	-0.064	0.036	0.006	35.994	1.562	0.582	0.969
LThickness	2907	-0.014	0.024	-0.061	0.033	0.004	30.355	1.436	0.550	0.969
RThickness	2907	-0.010	0.027	-0.063	0.042	0.008	43.677	1.775	0.699	0.969

Table S8: Cortical Surface Area Continuous Model - No Surface Area Covariate

Region	N	Effect Size (r)	SE	Lower CI	Upper CI	tau^2	I^2	H^2	pvalue	FDRp
L_bankssts	2682	0.009	0.019	-0.028	0.047	0.000	0.000	1.000	0.627	0.916
L_caudalanteriorcingulate	2812	-0.005	0.022	-0.048	0.039	0.002	19.198	1.238	0.834	0.936
L_caudalmiddlefrontal	2815	-0.009	0.021	-0.050	0.033	0.002	12.851	1.147	0.685	0.924
L_cuneus	2717	-0.018	0.020	-0.058	0.021	0.001	6.306	1.067	0.364	0.916
L_entorhinal	2683	-0.024	0.019	-0.063	0.014	0.000	1.631	1.017	0.209	0.916
L_fusiform	2798	-0.036	0.019	-0.072	0.001	0.000	0.000	1.000	0.059	0.916
L_inferiorparietal	2757	-0.007	0.019	-0.044	0.030	0.000	0.000	1.000	0.712	0.931
L_inferiortemporal	2771	-0.010	0.019	-0.047	0.027	0.000	0.000	1.000	0.590	0.916
L_isthmuscingulate	2826	0.008	0.019	-0.028	0.045	0.000	0.000	1.000	0.659	0.924
L_lateraloccipital	2794	-0.033	0.019	-0.070	0.003	0.000	0.000	1.000	0.075	0.916
L_lateralorbitofrontal	2801	-0.005	0.019	-0.042	0.032	0.000	0.000	1.000	0.783	0.936
L_lingual	2778	0.002	0.025	-0.048	0.052	0.005	34.053	1.516	0.932	0.949
L_medialorbitofrontal	2790	-0.027	0.032	-0.089	0.035	0.014	58.706	2.422	0.398	0.916
L_middletemporal	2691	0.013	0.019	-0.025	0.050	0.000	0.000	1.000	0.514	0.916
L_parahippocampal	2815	-0.051	0.019	-0.087	-0.014	0.000	0.000	1.000	0.007	0.253
L_paracentral	2825	0.021	0.028	-0.035	0.076	0.009	48.043	1.925	0.465	0.916
L_parsopercularis	2803	0.010	0.019	-0.026	0.047	0.000	0.000	1.000	0.580	0.916
L_parsorbitalis	2813	0.012	0.019	-0.025	0.049	0.000	0.000	1.000	0.521	0.916
L_parstriangularis	2801	-0.018	0.019	-0.055	0.019	0.000	0.000	1.000	0.344	0.916
L_pericalcarine	2751	-0.018	0.024	-0.065	0.028	0.003	24.680	1.328	0.439	0.916
L_postcentral	2771	0.015	0.019	-0.022	0.052	0.000	0.000	1.000	0.431	0.916

Region	N	Effect Size (r)	SE	Lower CI	Upper CI	tau^2	I^2	H^2	pvalue	FDRp
L_posteriorcingulate	2822	0.013	0.019	-0.024	0.049	0.000	0.000	1.000	0.504	0.916
L_precentral	2787	-0.017	0.019	-0.054	0.019	0.000	0.000	1.000	0.355	0.916
L_precuneus	2815	0.008	0.034	-0.059	0.074	0.018	64.406	2.809	0.823	0.936
L_rostralanteriorcingulate	2803	-0.020	0.020	-0.060	0.020	0.001	8.361	1.091	0.334	0.916
L_rostralmiddlefrontal	2814	0.009	0.019	-0.028	0.045	0.000	0.000	1.000	0.648	0.924
L_superiorfrontal	2807	0.003	0.029	-0.053	0.059	0.009	47.954	1.921	0.920	0.949
L_superiorparietal	2779	0.023	0.027	-0.030	0.076	0.007	41.086	1.697	0.398	0.916
L_superiortemporal	2665	0.008	0.023	-0.037	0.053	0.003	19.092	1.236	0.736	0.935
L_supramarginal	2705	0.033	0.026	-0.017	0.083	0.005	33.790	1.510	0.194	0.916
L_frontalpole	2830	-0.006	0.024	-0.054	0.042	0.005	31.018	1.450	0.800	0.936
L_temporalpole	2791	-0.024	0.023	-0.070	0.021	0.003	24.406	1.323	0.298	0.916
L_transversetemporal	2831	-0.016	0.019	-0.053	0.021	0.000	0.000	1.000	0.391	0.916
L_insula	2757	-0.021	0.024	-0.068	0.025	0.004	26.053	1.352	0.368	0.916
R_bankssts	2768	0.019	0.027	-0.035	0.073	0.008	43.014	1.755	0.486	0.916
R_caudalanteriorcingulate	2824	-0.017	0.019	-0.054	0.019	0.000	0.089	1.001	0.353	0.916
R_caudalmiddlefrontal	2812	-0.027	0.019	-0.063	0.010	0.000	0.000	1.000	0.156	0.916
R_cuneus	2731	-0.019	0.023	-0.065	0.027	0.003	23.123	1.301	0.413	0.916
R_entorhinal	2621	-0.042	0.029	-0.099	0.016	0.010	47.935	1.921	0.157	0.916
R_fusiform	2805	-0.005	0.019	-0.042	0.032	0.000	0.000	1.000	0.783	0.936
R_inferiorparietal	2758	0.000	0.019	-0.038	0.037	0.000	0.000	1.000	0.981	0.981
R_inferiortemporal	2783	-0.008	0.023	-0.052	0.036	0.003	20.114	1.252	0.726	0.934
R_isthmuscingulate	2817	0.030	0.029	-0.026	0.087	0.010	50.300	2.012	0.294	0.916
R_lateraloccipital	2796	-0.019	0.019	-0.056	0.018	0.000	0.000	1.000	0.322	0.916
R_lateralorbitofrontal	2781	-0.010	0.019	-0.047	0.027	0.000	0.000	1.000	0.587	0.916
R_lingual	2781	-0.016	0.019	-0.053	0.021	0.000	0.000	1.000	0.401	0.916
R_medialorbitofrontal	2775	-0.039	0.019	-0.076	-0.002	0.000	0.000	1.000	0.038	0.916
R_middletemporal	2762	-0.015	0.019	-0.053	0.022	0.000	0.000	1.000	0.415	0.916
R_parahippocampal	2825	-0.005	0.021	-0.046	0.036	0.001	10.837	1.122	0.806	0.936
R_paracentral	2822	-0.006	0.027	-0.059	0.047	0.008	42.734	1.746	0.830	0.936
R_parsopercularis	2787	0.012	0.019	-0.025	0.049	0.000	0.000	1.000	0.531	0.916
R_parsorbitalis	2810	-0.022	0.024	-0.068	0.024	0.004	25.931	1.350	0.348	0.916
R_parstriangularis	2781	-0.004	0.019	-0.041	0.033	0.000	0.000	1.000	0.842	0.936
R_pericalcarine	2735	-0.034	0.019	-0.072	0.003	0.000	0.000	1.000	0.072	0.916
R_postcentral	2785	0.028	0.022	-0.016	0.073	0.003	19.763	1.246	0.205	0.916

Region	N	Effect Size (r)	SE	Lower CI	Upper CI	tau^2	I^2	H^2	pvalue	FDRp
R_posteriorcingulate	2826	-0.005	0.019	-0.043	0.032	0.000	2.394	1.025	0.776	0.936
R_precentral	2791	-0.008	0.019	-0.045	0.029	0.000	0.000	1.000	0.684	0.924
R_precuneus	2814	0.034	0.031	-0.026	0.094	0.012	55.228	2.234	0.270	0.916
R_rostralanteriorcingulate	2781	-0.011	0.020	-0.051	0.029	0.001	8.245	1.090	0.592	0.916
R_rostralmiddlefrontal	2795	0.001	0.019	-0.036	0.038	0.000	0.000	1.000	0.967	0.976
R_superiorfrontal	2809	-0.006	0.024	-0.052	0.040	0.004	25.644	1.345	0.805	0.936
R_superiorparietal	2794	0.021	0.027	-0.031	0.074	0.007	40.321	1.676	0.425	0.916
R_superiortemporal	2730	0.003	0.019	-0.035	0.040	0.000	0.000	1.000	0.888	0.947
R_supramarginal	2723	-0.005	0.025	-0.054	0.044	0.005	31.945	1.469	0.850	0.936
R_frontalpole	2821	0.012	0.019	-0.025	0.048	0.000	0.000	1.000	0.534	0.916
R_temporalpole	2748	-0.009	0.019	-0.047	0.028	0.000	0.000	1.000	0.625	0.916
R_transversetemporal	2833	-0.013	0.022	-0.057	0.030	0.002	19.490	1.242	0.550	0.916
R_insula	2725	-0.010	0.019	-0.047	0.028	0.000	0.000	1.000	0.607	0.916
LSurfArea	2835	-0.012	0.019	-0.048	0.025	0.000	0.000	1.000	0.536	0.916
RSurfArea	2835	-0.010	0.019	-0.047	0.027	0.000	0.000	1.000	0.597	0.916

Table S9: Effect of Schizotypy in Subgroup with Smoking Data

Region	N	Effect Size (r)	SE	Lower CI	Upper CI	tau^2	I^2	H^2	pvalue	FDRp
L_bankssts	1212	-0.034	0.029	-0.090	0.022	0.000	0.000	1.000	0.239	0.958
L_caudalanteriorcingulate	1300	0.006	0.046	-0.084	0.096	0.009	56.059	2.276	0.902	0.960
L_caudalmiddlefrontal	1307	-0.013	0.075	-0.160	0.135	0.036	84.669	6.523	0.864	0.960
L_cuneus	1276	-0.010	0.049	-0.107	0.087	0.011	61.171	2.575	0.840	0.960
L_entorhinal	1233	-0.017	0.033	-0.082	0.049	0.002	19.491	1.242	0.614	0.958
L_fusiform	1311	-0.068	0.049	-0.163	0.028	0.011	61.796	2.618	0.166	0.958
L_inferiorparietal	1308	-0.034	0.043	-0.118	0.051	0.007	50.303	2.012	0.433	0.958
L_inferiortemporal	1293	-0.045	0.028	-0.099	0.010	0.000	0.000	1.000	0.109	0.958
L_isthmuscingulate	1312	0.004	0.041	-0.075	0.084	0.005	44.816	1.812	0.915	0.964
L_lateraloccipital	1313	-0.011	0.046	-0.102	0.080	0.009	57.545	2.355	0.819	0.960
L_lateralorbitofrontal	1315	-0.014	0.039	-0.089	0.062	0.004	39.168	1.644	0.724	0.958
L_lingual	1306	-0.011	0.032	-0.073	0.051	0.001	16.309	1.195	0.728	0.958
L_medialorbitofrontal	1303	0.037	0.029	-0.020	0.093	0.000	5.830	1.062	0.203	0.958
L_middletemporal	1250	-0.012	0.028	-0.067	0.043	0.000	0.000	1.000	0.668	0.958
L_parahippocampal	1313	-0.020	0.041	-0.101	0.060	0.006	45.539	1.836	0.616	0.958

[a] Sites with smoking data include BONNRISC, BONNSPQ, FOR2107-MR, FOR2017-MS, MNC, NYC, ZUR1, ZUR2

Region	N	Effect Size (r)	SE	Lower CI	Upper CI	tau^2	I^2	H^2	pvalue	FDRp
L_paracentral	1312	-0.049	0.063	-0.171	0.074	0.022	77.198	4.386	0.435	0.958
L_parsopercularis	1304	-0.053	0.049	-0.149	0.043	0.011	61.722	2.612	0.280	0.958
L_parsorbitalis	1309	-0.013	0.028	-0.067	0.042	0.000	0.000	1.000	0.649	0.958
L_parstriangularis	1307	-0.048	0.076	-0.196	0.101	0.037	84.997	6.665	0.531	0.958
L_pericalcarine	1291	0.006	0.041	-0.073	0.086	0.005	44.174	1.791	0.874	0.960
L_postcentral	1294	-0.064	0.071	-0.203	0.075	0.031	82.562	5.735	0.367	0.958
L_posteriorcingulate	1311	0.000	0.028	-0.054	0.054	0.000	0.000	1.000	0.997	0.997
L_precentral	1298	-0.028	0.044	-0.115	0.058	0.007	52.802	2.119	0.521	0.958
L_precuneus	1315	-0.035	0.029	-0.093	0.022	0.001	7.797	1.085	0.230	0.958
L_rostralanteriorcingulate	1298	0.023	0.028	-0.031	0.077	0.000	0.000	1.000	0.403	0.958
L_rostralmiddlefrontal	1309	0.001	0.028	-0.053	0.055	0.000	0.000	1.000	0.975	0.985
L_superiorfrontal	1300	-0.031	0.046	-0.121	0.059	0.009	56.317	2.289	0.504	0.958
L_superiorparietal	1306	-0.066	0.053	-0.170	0.038	0.014	67.730	3.099	0.211	0.958
L_superiortemporal	1214	-0.054	0.043	-0.138	0.030	0.006	47.254	1.896	0.209	0.958
L_supramarginal	1268	-0.041	0.042	-0.124	0.042	0.006	47.590	1.908	0.330	0.958
L_frontalpole	1315	0.037	0.057	-0.074	0.148	0.017	71.940	3.564	0.510	0.958
L_temporalpole	1312	0.014	0.046	-0.077	0.104	0.009	56.948	2.323	0.767	0.958
L_transversetemporal	1315	0.025	0.065	-0.103	0.153	0.025	79.204	4.809	0.701	0.958
L_insula	1277	0.001	0.028	-0.054	0.056	0.000	0.000	1.000	0.969	0.985
R_bankssts	1275	-0.045	0.028	-0.100	0.009	0.000	0.000	1.000	0.105	0.958
R_caudalanteriorcingulate	1310	0.040	0.062	-0.082	0.162	0.022	76.811	4.312	0.520	0.958
R_caudalmiddlefrontal	1312	-0.010	0.028	-0.064	0.044	0.000	0.000	1.000	0.713	0.958
R_cuneus	1283	-0.020	0.055	-0.128	0.088	0.015	69.577	3.287	0.717	0.958
R_entorhinal	1204	-0.019	0.049	-0.116	0.078	0.011	59.891	2.493	0.703	0.958
R_fusiform	1314	-0.051	0.033	-0.115	0.013	0.002	20.514	1.258	0.119	0.958
R_inferiorparietal	1304	-0.005	0.062	-0.127	0.116	0.021	76.427	4.242	0.929	0.968
R_inferiortemporal	1307	-0.034	0.041	-0.115	0.047	0.006	46.304	1.862	0.415	0.958
R_isthmuscingulate	1309	-0.046	0.028	-0.100	0.008	0.000	0.000	1.000	0.097	0.958
R_lateraloccipital	1311	-0.011	0.053	-0.114	0.092	0.013	67.023	3.032	0.834	0.960
R_lateralorbitofrontal	1312	-0.039	0.055	-0.148	0.069	0.016	70.208	3.357	0.476	0.958
R_lingual	1303	-0.021	0.050	-0.119	0.077	0.011	63.143	2.713	0.677	0.958
R_medialorbitofrontal	1303	0.061	0.027	0.007	0.115	0.000	0.000	1.000	0.027	0.958
R_middletemporal	1303	-0.029	0.028	-0.083	0.025	0.000	0.000	1.000	0.293	0.958

[a] Sites with smoking data include BONNRISC, BONNSPQ, FOR2107-MR, FOR2017-MS, MNC, NYC, ZUR1, ZUR2

Region	N	Effect Size (r)	SE	Lower CI	Upper CI	tau^2	I^2	H^2	pvalue	FDRp
R_parahippocampal	1317	-0.039	0.059	-0.154	0.077	0.019	74.005	3.847	0.511	0.958
R_paracentral	1309	-0.012	0.063	-0.135	0.112	0.022	77.599	4.464	0.854	0.960
R_parsopercularis	1306	0.005	0.041	-0.076	0.087	0.006	46.449	1.867	0.896	0.960
R_parsorbitalis	1313	-0.027	0.028	-0.081	0.027	0.000	0.000	1.000	0.323	0.958
R_parstriangularis	1302	-0.014	0.040	-0.094	0.065	0.005	43.765	1.778	0.721	0.958
R_pericalcarine	1279	0.009	0.047	-0.083	0.101	0.009	57.406	2.348	0.846	0.960
R_postcentral	1291	-0.062	0.039	-0.138	0.014	0.004	40.122	1.670	0.112	0.958
R_posteriorcingulate	1313	0.017	0.028	-0.037	0.071	0.000	0.000	1.000	0.533	0.958
R_precentral	1297	-0.047	0.054	-0.152	0.058	0.014	68.000	3.125	0.382	0.958
R_precuneus	1312	-0.027	0.055	-0.136	0.081	0.015	70.344	3.372	0.621	0.958
R_rostralanteriorcingulate	1289	0.019	0.041	-0.062	0.100	0.006	46.023	1.853	0.641	0.958
R_rostralmiddlefrontal	1309	-0.001	0.028	-0.055	0.053	0.000	0.000	1.000	0.965	0.985
R_superiorfrontal	1306	-0.015	0.044	-0.102	0.072	0.007	52.905	2.123	0.734	0.958
R_superiorparietal	1309	-0.055	0.052	-0.157	0.048	0.013	66.805	3.013	0.295	0.958
R_superiortemporal	1258	-0.047	0.059	-0.163	0.070	0.019	73.744	3.809	0.433	0.958
R_supramarginal	1278	-0.029	0.028	-0.084	0.025	0.000	0.000	1.000	0.293	0.958
R_frontalpole	1308	0.018	0.028	-0.036	0.072	0.000	0.000	1.000	0.520	0.958
R_temporalpole	1306	-0.007	0.045	-0.096	0.082	0.008	55.114	2.228	0.874	0.960
R_transversetemporal	1317	-0.042	0.044	-0.128	0.044	0.007	52.429	2.102	0.336	0.958
R_insula	1252	-0.023	0.065	-0.152	0.105	0.025	78.400	4.630	0.721	0.958
LThickness	1318	-0.054	0.060	-0.170	0.063	0.019	74.949	3.992	0.368	0.958
RThickness	1318	-0.053	0.057	-0.165	0.059	0.017	72.805	3.677	0.356	0.958
LFullSurf	1318	0.017	0.028	-0.037	0.071	0.000	0.000	1.000	0.545	0.958
RFullSurf	1318	0.013	0.028	-0.041	0.067	0.000	0.000	1.000	0.636	0.958

[a] Sites with smoking data include BONNRISC, BONNSPQ, FOR2107-MR, FOR2017-MS, MNC, NYC, ZUR1, ZUR2

Table S10: Effect of Schizopty Controlling for Smoking Status

Region	N	Effect Size (r)	SE	Lower CI	Upper CI	tau^2	I^2	H^2	pvalue	FDRp
L_bankssts	1201	-0.050	0.029	-0.106	0.006	0.000	0.000	1.000	0.082	0.921
L_caudalanteriorcingulate	1288	0.017	0.038	-0.058	0.093	0.004	37.894	1.610	0.651	0.965
L_caudalmiddlefrontal	1295	-0.004	0.072	-0.145	0.137	0.032	82.782	5.808	0.958	0.977
L_cuneus	1266	-0.010	0.049	-0.106	0.086	0.010	60.220	2.514	0.841	0.965

[a] Sites with smoking data include BONNRISC, BONNSPQ, FOR2107-MR, FOR2017-MS, MNC, NYC, ZUR1, ZUR2

Region	N	Effect Size (r)	SE	Lower CI	Upper CI	tau^2	I^2	H^2	pvalue	FDRp
L_entorhinal	1221	-0.023	0.035	-0.092	0.045	0.002	24.152	1.318	0.504	0.965
L_fusiform	1299	-0.062	0.043	-0.146	0.022	0.007	49.546	1.982	0.145	0.965
L_inferiorparietal	1296	-0.020	0.028	-0.075	0.034	0.000	0.000	1.000	0.462	0.965
L_inferiortemporal	1281	-0.049	0.028	-0.103	0.006	0.000	0.000	1.000	0.082	0.921
L_isthmuscingulate	1300	-0.020	0.037	-0.092	0.052	0.003	32.997	1.492	0.578	0.965
L_lateraloccipital	1301	-0.021	0.048	-0.116	0.074	0.010	60.491	2.531	0.661	0.965
L_lateralorbitofrontal	1303	-0.004	0.041	-0.084	0.076	0.005	44.674	1.807	0.925	0.965
L_lingual	1295	-0.014	0.035	-0.083	0.056	0.003	28.634	1.401	0.701	0.965
L_medialorbitofrontal	1291	0.046	0.028	-0.008	0.100	0.000	0.000	1.000	0.095	0.921
L_middletemporal	1238	-0.017	0.028	-0.072	0.039	0.000	0.033	1.000	0.549	0.965
L_parahippocampal	1302	-0.009	0.028	-0.063	0.046	0.000	0.000	1.000	0.754	0.965
L_paracentral	1300	-0.042	0.061	-0.162	0.078	0.020	75.676	4.111	0.494	0.965
L_parsopercularis	1292	-0.048	0.034	-0.116	0.019	0.002	25.734	1.347	0.162	0.965
L_parsorbitalis	1297	-0.007	0.028	-0.062	0.047	0.000	0.000	1.000	0.792	0.965
L_parstriangularis	1295	-0.038	0.072	-0.179	0.104	0.032	83.008	5.885	0.603	0.965
L_pericalcarine	1279	0.005	0.043	-0.080	0.090	0.007	49.744	1.990	0.913	0.965
L_postcentral	1283	-0.054	0.066	-0.184	0.076	0.025	79.353	4.843	0.414	0.965
L_posteriorcingulate	1299	0.003	0.028	-0.052	0.057	0.000	0.000	1.000	0.924	0.965
L_precentral	1286	-0.015	0.036	-0.085	0.055	0.003	29.552	1.419	0.673	0.965
L_precuneus	1303	-0.031	0.028	-0.085	0.023	0.000	0.000	1.000	0.259	0.965
L_rostralanteriorcingulate	1286	0.025	0.028	-0.030	0.079	0.000	0.000	1.000	0.376	0.965
L_rostralmiddlefrontal	1297	0.005	0.028	-0.050	0.059	0.000	0.000	1.000	0.864	0.965
L_superiorfrontal	1288	-0.014	0.039	-0.090	0.062	0.004	38.667	1.630	0.720	0.965
L_superiorparietal	1295	-0.034	0.036	-0.104	0.036	0.003	30.189	1.432	0.344	0.965
L_superiortemporal	1204	-0.048	0.033	-0.113	0.017	0.002	17.319	1.209	0.148	0.965
L_supramarginal	1257	-0.032	0.028	-0.087	0.024	0.000	0.000	1.000	0.263	0.965
L_frontalpole	1303	0.042	0.055	-0.066	0.150	0.015	69.699	3.300	0.442	0.965
L_temporalpole	1300	0.006	0.046	-0.084	0.096	0.008	55.975	2.271	0.897	0.965
L_transversetemporal	1303	0.021	0.066	-0.109	0.150	0.025	79.424	4.860	0.756	0.965
L_insula	1265	0.014	0.028	-0.041	0.069	0.000	0.000	1.000	0.624	0.965
R_bankssts	1263	-0.046	0.028	-0.101	0.009	0.000	0.000	1.000	0.099	0.921
R_caudalanteriorcingulate	1298	0.064	0.052	-0.038	0.165	0.013	65.301	2.882	0.219	0.965
R_caudalmiddlefrontal	1300	-0.001	0.028	-0.055	0.053	0.000	0.000	1.000	0.970	0.977

[a] Sites with smoking data include BONNRISC, BONNSPQ, FOR2107-MR, FOR2017-MS, MNC, NYC, ZUR1, ZUR2

Region	N	Effect Size (r)	SE	Lower CI	Upper CI	tau^2	I^2	H^2	pvalue	FDRp
R_cuneus	1272	-0.037	0.059	-0.152	0.078	0.018	72.731	3.667	0.532	0.965
R_entorhinal	1194	-0.018	0.051	-0.117	0.081	0.011	61.223	2.579	0.724	0.965
R_fusiform	1302	-0.048	0.028	-0.102	0.006	0.000	0.000	1.000	0.080	0.921
R_inferiorparietal	1292	0.005	0.052	-0.096	0.106	0.012	64.730	2.835	0.926	0.965
R_inferiortemporal	1295	-0.030	0.043	-0.113	0.054	0.006	48.554	1.944	0.482	0.965
R_isthmuscingulate	1297	-0.051	0.028	-0.105	0.003	0.000	0.000	1.000	0.067	0.921
R_lateraloccipital	1299	-0.018	0.050	-0.117	0.080	0.011	62.851	2.692	0.717	0.965
R_lateralorbitofrontal	1300	-0.025	0.052	-0.127	0.077	0.013	65.511	2.899	0.634	0.965
R_lingual	1292	-0.021	0.052	-0.123	0.081	0.013	65.559	2.904	0.683	0.965
R_medialorbitofrontal	1291	0.076	0.028	0.022	0.130	0.000	0.000	1.000	0.006	0.408
R_middletemporal	1291	-0.024	0.028	-0.078	0.030	0.000	0.000	1.000	0.389	0.965
R_parahippocampal	1305	-0.012	0.050	-0.110	0.085	0.011	62.527	2.669	0.804	0.965
R_paracentral	1297	0.002	0.054	-0.104	0.109	0.014	68.688	3.194	0.969	0.977
R_parsopercularis	1294	0.013	0.036	-0.057	0.084	0.003	30.281	1.434	0.712	0.965
R_parsorbitalis	1301	-0.024	0.028	-0.079	0.030	0.000	0.000	1.000	0.377	0.965
R_parstriangularis	1290	-0.022	0.044	-0.108	0.063	0.007	50.870	2.035	0.609	0.965
R_pericalcarine	1267	0.008	0.051	-0.091	0.107	0.012	62.691	2.680	0.872	0.965
R_postcentral	1279	-0.057	0.038	-0.131	0.016	0.004	35.290	1.545	0.127	0.965
R_posteriorcingulate	1301	0.027	0.028	-0.027	0.081	0.000	0.000	1.000	0.324	0.965
R_precentral	1285	-0.036	0.041	-0.116	0.044	0.005	43.598	1.773	0.376	0.965
R_precuneus	1300	-0.027	0.052	-0.129	0.074	0.013	65.343	2.885	0.599	0.965
R_rostralanteriorcingulate	1277	0.028	0.042	-0.054	0.110	0.006	46.562	1.871	0.505	0.965
R_rostralmiddlefrontal	1297	0.004	0.028	-0.050	0.058	0.000	0.000	1.000	0.878	0.965
R_superiorfrontal	1294	-0.014	0.049	-0.111	0.083	0.011	61.596	2.604	0.773	0.965
R_superiorparietal	1297	-0.040	0.039	-0.116	0.035	0.004	38.682	1.631	0.298	0.965
R_superiortemporal	1247	-0.024	0.048	-0.118	0.071	0.010	58.647	2.418	0.621	0.965
R_supramarginal	1266	-0.022	0.028	-0.077	0.033	0.000	0.000	1.000	0.440	0.965
R_frontalpole	1296	0.017	0.028	-0.037	0.072	0.000	0.000	1.000	0.538	0.965
R_temporalpole	1294	-0.008	0.045	-0.097	0.081	0.008	54.394	2.193	0.864	0.965
R_transversetemporal	1305	-0.028	0.036	-0.098	0.042	0.003	29.754	1.424	0.435	0.965
R_insula	1240	-0.011	0.066	-0.140	0.118	0.025	78.244	4.596	0.870	0.965
LThickness	1306	-0.042	0.051	-0.142	0.057	0.012	64.337	2.804	0.404	0.965
RThickness	1306	-0.040	0.050	-0.137	0.057	0.011	62.465	2.664	0.419	0.965

[a] Sites with smoking data include BONNRISC, BONNSPQ, FOR2107-MR, FOR2017-MS, MNC, NYC, ZUR1, ZUR2

Region	N	Effect Size (r)	SE	Lower CI	Upper CI	tau^2	I^2	H^2	pvalue	FDRp
LFullSurf	1306	0.018	0.028	-0.036	0.073	0.000	0.000	1.000	0.504	0.965
RFullSurf	1306	0.013	0.028	-0.041	0.067	0.000	0.000	1.000	0.639	0.965

[a] Sites with smoking data include BONNRISC, BONNSPQ, FOR2107-MR, FOR2017-MS, MNC, NYC, ZUR1, ZUR2

Table S11: Effect of Schizotypy in Subgroup with Smoking Data

Region	N	Effect Size (r)	SE	Lower CI	Upper CI	tau^2	I^2	H^2	pvalue	FDRp
LLatVent	1334	0.023	0.039	-0.053	0.100	0.005	42.665	1.744	0.550	0.877
RLatVent	1334	0.006	0.042	-0.076	0.089	0.006	49.716	1.989	0.885	0.996
Lthal	1325	0.004	0.066	-0.126	0.134	0.026	80.206	5.052	0.954	0.996
Lcaud	1326	0.035	0.044	-0.051	0.122	0.007	53.415	2.147	0.421	0.877
Lput	1292	0.003	0.031	-0.059	0.064	0.001	15.079	1.178	0.934	0.996
Lpal	1223	-0.035	0.029	-0.092	0.021	0.000	0.000	1.000	0.215	0.877
Lhippo	1326	0.016	0.041	-0.063	0.095	0.005	45.463	1.834	0.692	0.877
Lamyg	1320	0.025	0.027	-0.029	0.079	0.000	0.000	1.000	0.364	0.877
Laccumb	1318	0.017	0.042	-0.066	0.099	0.006	48.804	1.953	0.694	0.877
Rthal	1334	0.050	0.069	-0.084	0.185	0.029	81.830	5.504	0.464	0.877
Rcaud	1330	0.027	0.052	-0.075	0.129	0.013	66.927	3.024	0.607	0.877
Rput	1316	-0.008	0.033	-0.072	0.056	0.002	20.288	1.255	0.802	0.962
Rpal	1315	-0.035	0.043	-0.119	0.048	0.007	50.506	2.020	0.408	0.877
Rhippo	1329	0.023	0.030	-0.036	0.082	0.001	12.474	1.143	0.445	0.877
Ramyg	1315	0.031	0.027	-0.023	0.085	0.000	0.000	1.000	0.256	0.877
Raccumb	1319	0.024	0.052	-0.078	0.126	0.013	66.713	3.004	0.649	0.877

[a] Sites with smoking data include BONNRISC, BONNSPQ, FOR2107-MR, FOR2017-MS, MNC, NYC, ZUR1, ZUR2

Table S12: Effect of Schizopty Controlling for Smoking Status

Region	N	Effect Size (r)	SE	Lower CI	Upper CI	tau^2	I^2	H^2	pvalue	FDRp
LLatVent	1137	0.004	0.037	-0.069	0.077	0.002	25.333	1.339	0.914	0.962
RLatVent	1137	0.014	0.056	-0.095	0.123	0.012	64.227	2.795	0.799	0.962

[a] Sites with smoking data include BONNRISC, BONNSPQ, FOR2107-MR, FOR2017-MS, MNC, NYC, ZUR1, ZUR2

Region	N	Effect Size (r)	SE	Lower CI	Upper CI	tau^2	I^2	H^2	pvalue	FDRp
Lthal	1137	0.004	0.082	-0.157	0.165	0.037	84.505	6.454	0.962	0.962
Lcaud	1129	0.028	0.048	-0.065	0.122	0.007	50.868	2.035	0.554	0.962
Lput	1098	-0.019	0.030	-0.078	0.040	0.000	0.000	1.000	0.530	0.962
Lpal	1034	-0.030	0.031	-0.091	0.031	0.000	0.000	1.000	0.333	0.962
Lhippo	1132	-0.008	0.030	-0.066	0.051	0.000	0.000	1.000	0.794	0.962
Lamyg	1127	-0.003	0.030	-0.061	0.056	0.000	0.000	1.000	0.926	0.962
Laccumb	1123	0.009	0.050	-0.090	0.107	0.009	55.603	2.252	0.859	0.962
Rthal	1137	0.059	0.088	-0.114	0.233	0.045	86.955	7.666	0.501	0.962
Rcaud	1133	0.015	0.057	-0.097	0.127	0.013	65.786	2.923	0.797	0.962
Rput	1120	-0.036	0.030	-0.095	0.023	0.000	0.000	1.000	0.228	0.962
Rpal	1119	-0.036	0.037	-0.107	0.036	0.002	22.917	1.297	0.329	0.962
Rhippo	1136	-0.031	0.030	-0.089	0.027	0.000	0.000	1.000	0.295	0.962
Ramyg	1122	0.014	0.030	-0.044	0.072	0.000	0.000	1.000	0.641	0.962
Raccumb	1124	0.014	0.050	-0.084	0.112	0.009	55.242	2.234	0.781	0.962

[a] Sites with smoking data include BONNRISC, BONNSPQ, FOR2107-MR, FOR2017-MS, MNC, NYC, ZUR1, ZUR2

Table S13: Schizotypy Questionaire Moderator

Region	Q	pvalue	df
L_bankssts	0.1210944	0.7278506	1
L_caudalanteriorcingulate	0.0074802	0.9310785	1
L_caudalmiddlefrontal	0.1761127	0.6747354	1
L_cuneus	0.5012191	0.4789649	1
L_entorhinal	0.0052265	0.9423674	1
L_fusiform	0.5831693	0.4450727	1
L_inferiorparietal	0.1734402	0.6770723	1
L_inferiortemporal	0.0547494	0.8149959	1
L_isthmuscingulate	0.0493176	0.8242549	1
L_lateraloccipital	0.0250159	0.8743275	1
L_lateralorbitofrontal	4.9187359	0.0265669	1
L_lingual	0.1367613	0.7115221	1
L_medialorbitofrontal	0.1520148	0.6966173	1
L_middletemporal	0.1771360	0.6738461	1
L_parahippocampal	1.7025615	0.1919533	1

[a] Uncorrected p-values. All results were non-significant (all pFDR >.05) after FDR correction.

Region	Q	pvalue	df
L_paracentral	0.0618448	0.8036036	1
L_parsopercularis	0.0890043	0.7654469	1
L_parsorbitalis	0.0007130	0.9786968	1
L_parstriangularis	0.0397984	0.8418753	1
L_pericalcarine	0.2736839	0.6008712	1
L_postcentral	0.3397632	0.5599660	1
L_posteriorcingulate	0.4695531	0.4931928	1
L_precentral	0.1011104	0.7505012	1
L_precuneus	0.7395842	0.3897941	1
L_rostralanteriorcingulate	0.5078078	0.4760894	1
L_rostralmiddlefrontal	0.1179889	0.7312260	1
L_superiorfrontal	0.0942487	0.7588437	1
L_superiorparietal	0.0020676	0.9637321	1
L_superiortemporal	1.4838901	0.2231669	1
L_supramarginal	0.0085677	0.9262515	1
L_frontalpole	0.1505594	0.6980014	1
L_temporalpole	1.3129276	0.2518653	1
L_transversetemporal	0.0342318	0.8532146	1
L_insula	0.0760752	0.7826883	1
R_bankssts	0.0178818	0.8936217	1
R_caudalanteriorcingulate	3.0392794	0.0812720	1
R_caudalmiddlefrontal	1.4702307	0.2253093	1
R_cuneus	0.7697907	0.3802818	1
R_entorhinal	0.0016672	0.9674300	1
R_fusiform	0.8415015	0.3589677	1
R_inferiorparietal	0.4163542	0.5187618	1
R_inferiortemporal	0.0284832	0.8659777	1
R_isthmuscingulate	2.4462291	0.1178076	1
R_lateraloccipital	0.0014794	0.9693182	1
R_lateralorbitofrontal	0.0004730	0.9826490	1
R_lingual	0.0499329	0.8231800	1
R_medialorbitofrontal	0.4599144	0.4976640	1
R_middletemporal	0.3277040	0.5670142	1

[a] Uncorrected p-values. All results were non-significant (all pFDR >.05) after FDR correction.

Region	Q	pvalue	df
R_parahippocampal	0.0794971	0.7779801	1
R_paracentral	1.0389130	0.3080744	1
R_parsopercularis	0.4811284	0.4879116	1
R_parsorbitalis	0.7505329	0.3863076	1
R_parstriangularis	0.3795819	0.5378271	1
R_pericalcarine	0.0985043	0.7536319	1
R_postcentral	1.1597696	0.2815133	1
R_posteriorcingulate	0.1642859	0.6852409	1
R_precentral	0.5599779	0.4542691	1
R_precuneus	0.4096600	0.5221421	1
R_rostralanteriorcingulate	0.3231411	0.5697261	1
R_rostralmiddlefrontal	0.1737071	0.6768380	1
R_superiorfrontal	0.4339876	0.5100387	1
R_superiorparietal	1.4224680	0.2329976	1
R_superiortemporal	0.2801484	0.5966039	1
R_supramarginal	0.3026792	0.5822077	1
R_frontalpole	1.8713180	0.1713242	1
R_temporalpole	0.5870206	0.4435736	1
R_transversetemporal	0.1103299	0.7397689	1
R_insula	0.3142672	0.5750734	1
LThickness	0.0512076	0.8209751	1
RThickness	0.3369752	0.5615804	1
LFullSurf	0.5240306	0.4691274	1
RFullSurf	1.7808076	0.1820502	1

[a] Uncorrected p-values. All results were non-significant (all pFDR >.05) after FDR correction.

Table S14: Scanner Field Strength Moderator

Region	Q	pvalue	df
L_bankssts	0.0006394	0.9798269	1
L_caudalanteriorcingulate	0.2951084	0.5869654	1
L_caudalmiddlefrontal	0.2167744	0.6415087	1
L_cuneus	4.2191209	0.0399709	1
L_entorhinal	0.0104878	0.9184313	1

[a] Uncorrected p-values. All results were non-significant (all pFDR >.05) after FDR correction.

Region	Q	pvalue	df
L_fusiform	0.1792749	0.6719971	1
L_inferiorparietal	0.0000107	0.9973896	1
L_inferiortemporal	3.0161160	0.0824407	1
L_isthmuscingulate	2.5115878	0.1130120	1
L_lateraloccipital	1.4691143	0.2254855	1
L_lateralorbitofrontal	0.6764936	0.4107968	1
L_lingual	0.0012561	0.9717282	1
L_medialorbitofrontal	0.2222077	0.6373629	1
L_middletemporal	12.7054879	0.0003646	1
L_parahippocampal	4.7644181	0.0290539	1
L_paracentral	0.0021468	0.9630445	1
L_parsopercularis	0.0235230	0.8781047	1
L_parsorbitalis	1.6440908	0.1997651	1
L_parstriangularis	0.1669503	0.6828365	1
L_pericalcarine	0.5758518	0.4479429	1
L_postcentral	5.9894980	0.0143913	1
L_posteriorcingulate	0.0137002	0.9068223	1
L_precentral	0.2194271	0.6394768	1
L_precuneus	0.1527921	0.6958813	1
L_rostralanteriorcingulate	0.4284796	0.5127359	1
L_rostralmiddlefrontal	1.4619095	0.2266265	1
L_superiorfrontal	0.0059823	0.9383488	1
L_superiorparietal	0.5162971	0.4724255	1
L_superiortemporal	0.8173949	0.3659429	1
L_supramarginal	0.6481216	0.4207850	1
L_frontalpole	0.6506901	0.4198661	1
L_temporalpole	0.0805663	0.7765314	1
L_transversetemporal	1.0427502	0.3071827	1
L_insula	0.1212956	0.7276336	1
R_bankssts	0.8296194	0.3623826	1
R_caudalanteriorcingulate	0.0989807	0.7530563	1
R_caudalmiddlefrontal	1.1570111	0.2820863	1
R_cuneus	1.2530542	0.2629699	1

[a] Uncorrected p-values. All results were non-significant (all pFDR >.05) after FDR correction.

Region	Q	pvalue	df
R_entorhinal	0.0021527	0.9629933	1
R_fusiform	0.3973368	0.5284679	1
R_inferiorparietal	0.0333550	0.8550855	1
R_inferiortemporal	0.1584371	0.6905995	1
R_isthmuscingulate	6.6778748	0.0097617	1
R_lateraloccipital	0.2195640	0.6393723	1
R_lateralorbitofrontal	0.7139524	0.3981348	1
R_lingual	1.2770981	0.2584393	1
R_medialorbitofrontal	0.3690545	0.5435201	1
R_middletemporal	0.6875686	0.4069926	1
R_parahippocampal	14.7253803	0.0001244	1
R_paracentral	0.0709597	0.7899445	1
R_parsopercularis	0.9496323	0.3298129	1
R_parsorbitalis	2.2656379	0.1322717	1
R_parstriangularis	0.1007702	0.7509073	1
R_pericalcarine	0.6426488	0.4227533	1
R_postcentral	0.5802581	0.4462112	1
R_posteriorcingulate	6.7674999	0.0092833	1
R_precentral	0.0011865	0.9725223	1
R_precuneus	0.2293018	0.6320420	1
R_rostralanteriorcingulate	0.2940208	0.5876554	1
R_rostralmiddlefrontal	3.2650897	0.0707692	1
R_superiorfrontal	0.3575370	0.5498773	1
R_superiorparietal	0.1896814	0.6631819	1
R_superiortemporal	0.0927115	0.7607580	1
R_supramarginal	0.6270878	0.4284255	1
R_frontalpole	0.0470704	0.8282418	1
R_temporalpole	0.3282038	0.5667187	1
R_transversetemporal	0.0315378	0.8590459	1
R_insula	0.0007913	0.9775582	1
LThickness	0.0846771	0.7710564	1
RThickness	0.1418164	0.7064819	1
LFullSurf	0.0004285	0.9834853	1

[a] Uncorrected p-values. All results were non-significant (all pFDR >.05) after FDR correction.

Region	Q	pvalue	df
RFullSurf	0.1010385	0.7505869	1

[a] Uncorrected p-values. All results were non-significant (all pFDR >.05) after FDR correction.

Table S15: Scanner Number Moderator

Region	Q	pvalue	df
L_bankssts	0.0017196	0.9669223	1
L_caudalanteriorcingulate	0.2855258	0.5931020	1
L_caudalmiddlefrontal	0.0159449	0.8995158	1
L_cuneus	1.7940219	0.1804369	1
L_entorhinal	0.5064899	0.4766623	1
L_fusiform	0.0155664	0.9007093	1
L_inferiorparietal	0.0091129	0.9239485	1
L_inferiortemporal	1.8208068	0.1772173	1
L_isthmuscingulate	0.7260419	0.3941692	1
L_lateraloccipital	0.7061612	0.4007212	1
L_lateralorbitofrontal	0.6465127	0.4213623	1
L_lingual	0.0221572	0.8816696	1
L_medialorbitofrontal	0.0599806	0.8065267	1
L_middletemporal	2.5910831	0.1074668	1
L_parahippocampal	1.7577107	0.1849103	1
L_paracentral	0.9833386	0.3213759	1
L_parsopercularis	0.0287153	0.8654380	1
L_parsorbitalis	1.9717844	0.1602586	1
L_parstriangularis	0.0790648	0.7785687	1
L_pericalcarine	0.2553202	0.6133536	1
L_postcentral	0.7877598	0.3747784	1
L_posteriorcingulate	0.0759918	0.7828045	1
L_precentral	0.2840249	0.5940752	1
L_precuneus	1.8770804	0.1706664	1
L_rostralanteriorcingulate	2.0579533	0.1514134	1
L_rostralmiddlefrontal	0.1207963	0.7281724	1
L_superiorfrontal	0.0120182	0.9127046	1
L_superiorparietal	0.0055395	0.9406702	1

[a] Uncorrected p-values. All results were non-significant (all pFDR >.05) after FDR correction.

Region	Q	pvalue	df
L_superiortemporal	0.0800926	0.7771720	1
L_supramarginal	2.2609348	0.1326740	1
L_frontalpole	0.8267064	0.3632266	1
L_temporalpole	0.3910813	0.5317316	1
L_transversetemporal	0.0209110	0.8850217	1
L_insula	2.1858419	0.1392849	1
R_bankssts	1.5433915	0.2141137	1
R_caudalanteriorcingulate	0.2579515	0.6115310	1
R_caudalmiddlefrontal	1.8352235	0.1755118	1
R_cuneus	0.8475660	0.3572418	1
R_entorhinal	0.1257431	0.7228872	1
R_fusiform	0.5220107	0.4699852	1
R_inferiorparietal	1.7477582	0.1861588	1
R_inferiortemporal	0.0096758	0.9216418	1
R_isthmuscingulate	0.3380831	0.5609378	1
R_lateraloccipital	2.4199666	0.1197975	1
R_lateralorbitofrontal	0.7684165	0.3807074	1
R_lingual	1.3826518	0.2396500	1
R_medialorbitofrontal	0.0545302	0.8153600	1
R_middletemporal	0.3064360	0.5798755	1
R_parahippocampal	10.6294360	0.0011130	1
R_paracentral	0.6496865	0.4202248	1
R_parsopercularis	2.7693669	0.0960842	1
R_parsorbitalis	0.4203147	0.5167800	1
R_parstriangularis	0.3428816	0.5581706	1
R_pericalcarine	0.6571204	0.4175784	1
R_postcentral	2.0449024	0.1527168	1
R_posteriorcingulate	0.2225212	0.6371256	1
R_precentral	1.6571356	0.1979905	1
R_precuneus	0.1327091	0.7156395	1
R_rostralanteriorcingulate	0.0966012	0.7559470	1
R_rostralmiddlefrontal	0.1302150	0.7182092	1
R_superiorfrontal	0.4413176	0.5064872	1

[a] Uncorrected p-values. All results were non-significant (all pFDR >.05) after FDR correction.

Region	Q	pvalue	df
R_superiorparietal	0.1617291	0.6875696	1
R_superiortemporal	1.6381920	0.2005737	1
R_supramarginal	0.0491681	0.8245172	1
R_frontalpole	0.0188587	0.8907723	1
R_temporalpole	0.7669973	0.3811476	1
R_transversetemporal	1.2202363	0.2693143	1
R_insula	0.2308803	0.6308719	1
LThickness	0.4583218	0.4984093	1
RThickness	0.5620012	0.4534550	1
LFullSurf	0.8720794	0.3503803	1
RFullSurf	1.0719482	0.3005058	1

[a] Uncorrected p-values. All results were non-significant (all pFDR >.05) after FDR correction.

Table S16: FreeSurfer Version Moderator

Region	Q	pvalue	df
L_bankssts	0.0006394	0.9798269	1
L_caudalanteriorcingulate	0.2951084	0.5869654	1
L_caudalmiddlefrontal	0.2167744	0.6415087	1
L_cuneus	4.2191209	0.0399709	1
L_entorhinal	0.0104878	0.9184313	1
L_fusiform	0.1792749	0.6719971	1
L_inferiorparietal	0.0000107	0.9973896	1
L_inferiortemporal	3.0161160	0.0824407	1
L_isthmuscingulate	2.5115878	0.1130120	1
L_lateraloccipital	1.4691143	0.2254855	1
L_lateralorbitofrontal	0.6764936	0.4107968	1
L_lingual	0.0012561	0.9717282	1
L_medialorbitofrontal	0.2222077	0.6373629	1
L_middletemporal	12.7054879	0.0003646	1
L_parahippocampal	4.7644181	0.0290539	1
L_paracentral	0.0021468	0.9630445	1
L_parsopercularis	0.0235230	0.8781047	1
L_parsorbitalis	1.6440908	0.1997651	1

[a] Uncorrected p-values. All results were non-significant (all pFDR >.05) after FDR correction.

Region	Q	pvalue	df
L_parstriangularis	0.1669503	0.6828365	1
L_pericalcarine	0.5758518	0.4479429	1
L_postcentral	5.9894980	0.0143913	1
L_posteriorcingulate	0.0137002	0.9068223	1
L_precentral	0.2194271	0.6394768	1
L_precuneus	0.1527921	0.6958813	1
L_rostralanteriorcingulate	0.4284796	0.5127359	1
L_rostralmiddlefrontal	1.4619095	0.2266265	1
L_superiorfrontal	0.0059823	0.9383488	1
L_superiorparietal	0.5162971	0.4724255	1
L_superiortemporal	0.8173949	0.3659429	1
L_supramarginal	0.6481216	0.4207850	1
L_frontalpole	0.6506901	0.4198661	1
L_temporalpole	0.0805663	0.7765314	1
L_transversetemporal	1.0427502	0.3071827	1
L_insula	0.1212956	0.7276336	1
R_bankssts	0.8296194	0.3623826	1
R_caudalanteriorcingulate	0.0989807	0.7530563	1
R_caudalmiddlefrontal	1.1570111	0.2820863	1
R_cuneus	1.2530542	0.2629699	1
R_entorhinal	0.0021527	0.9629933	1
R_fusiform	0.3973368	0.5284679	1
R_inferiorparietal	0.0333550	0.8550855	1
R_inferiortemporal	0.1584371	0.6905995	1
R_isthmuscingulate	6.6778748	0.0097617	1
R_lateraloccipital	0.2195640	0.6393723	1
R_lateralorbitofrontal	0.7139524	0.3981348	1
R_lingual	1.2770981	0.2584393	1
R_medialorbitofrontal	0.3690545	0.5435201	1
R_middletemporal	0.6875686	0.4069926	1
R_parahippocampal	14.7253803	0.0001244	1
R_paracentral	0.0709597	0.7899445	1
R_parsopercularis	0.9496323	0.3298129	1

[a] Uncorrected p-values. All results were non-significant (all pFDR >.05) after FDR correction.

Region	Q	pvalue	df
R_parsorbitalis	2.2656379	0.1322717	1
R_parstriangularis	0.1007702	0.7509073	1
R_pericalcarine	0.6426488	0.4227533	1
R_postcentral	0.5802581	0.4462112	1
R_posteriorcingulate	6.7674999	0.0092833	1
R_precentral	0.0011865	0.9725223	1
R_precuneus	0.2293018	0.6320420	1
R_rostralanteriorcingulate	0.2940208	0.5876554	1
R_rostralmiddlefrontal	3.2650897	0.0707692	1
R_superiorfrontal	0.3575370	0.5498773	1
R_superiorparietal	0.1896814	0.6631819	1
R_superiortemporal	0.0927115	0.7607580	1
R_supramarginal	0.6270878	0.4284255	1
R_frontalpole	0.0470704	0.8282418	1
R_temporalpole	0.3282038	0.5667187	1
R_transversetemporal	0.0315378	0.8590459	1
R_insula	0.0007913	0.9775582	1
LThickness	0.0846771	0.7710564	1
RThickness	0.1418164	0.7064819	1
LFullSurf	0.0004285	0.9834853	1
RFullSurf	0.1010385	0.7505869	1

[a] Uncorrected p-values. All results were non-significant (all pFDR >.05) after FDR correction.

Table S17: Cortical Thickness Continuous Model

Region	N	Effect Size (r)	SE	Lower CI	Upper CI	tau^2	I^2	H^2	pvalue	FDRp
L_bankssts	2746	0.019	0.031	-0.041	0.079	0.012	54.381	2.192	0.5310	0.981
L_caudalanteriorcingulate	2884	0.019	0.019	-0.018	0.055	0.000	0.000	1.000	0.3090	0.910
L_caudalmiddlefrontal	2886	0.007	0.034	-0.060	0.073	0.018	65.196	2.873	0.8400	0.981
L_cuneus	2777	0.002	0.023	-0.044	0.048	0.003	24.046	1.317	0.9360	0.981
L_entorhinal	2755	-0.019	0.019	-0.057	0.018	0.000	0.000	1.000	0.3080	0.910
L_fusiform	2870	-0.038	0.020	-0.078	0.002	0.001	9.521	1.105	0.0620	0.555
L_inferiorparietal	2828	-0.004	0.019	-0.041	0.033	0.000	0.000	1.000	0.8360	0.981
L_inferiortemporal	2843	-0.003	0.019	-0.039	0.034	0.000	0.000	1.000	0.8840	0.981

Region	N	Effect Size (r)	SE	Lower CI	Upper CI	tau^2	I^2	H^2	pvalue	FDRp
L_isthmuscingulate	2898	-0.023	0.028	-0.077	0.032	0.009	47.412	1.902	0.4140	0.981
L_lateraloccipital	2866	0.035	0.023	-0.010	0.080	0.003	23.738	1.311	0.1240	0.844
L_lateralorbitofrontal	2873	0.006	0.024	-0.042	0.054	0.005	31.913	1.469	0.8020	0.981
L_lingual	2842	-0.013	0.019	-0.050	0.024	0.000	1.786	1.018	0.4870	0.981
L_medialorbitofrontal	2862	0.057	0.020	0.018	0.096	0.001	8.255	1.090	0.0040	0.120
L_middletemporal	2756	-0.002	0.030	-0.061	0.057	0.011	52.553	2.108	0.9430	0.981
L_parahippocampal	2888	0.012	0.028	-0.042	0.066	0.008	45.968	1.851	0.6580	0.981
L_paracentral	2897	-0.046	0.022	-0.089	-0.003	0.002	19.606	1.244	0.0350	0.477
L_parsopercularis	2875	-0.012	0.019	-0.048	0.025	0.000	0.000	1.000	0.5280	0.981
L_parsorbitalis	2885	0.010	0.019	-0.026	0.046	0.000	0.000	1.000	0.5910	0.981
L_parstriangularis	2873	-0.012	0.032	-0.075	0.051	0.015	60.455	2.529	0.7160	0.981
L_pericalcarine	2811	-0.019	0.026	-0.070	0.032	0.006	37.466	1.599	0.4700	0.981
L_postcentral	2838	-0.004	0.022	-0.047	0.039	0.002	17.479	1.212	0.8610	0.981
L_posteriorcingulate	2896	0.027	0.018	-0.009	0.064	0.000	0.000	1.000	0.1400	0.844
L_precentral	2855	-0.010	0.019	-0.046	0.027	0.000	0.000	1.000	0.5920	0.981
L_precuneus	2887	0.001	0.022	-0.042	0.044	0.002	18.010	1.220	0.9570	0.981
L_rostralanteriorcingulate	2875	0.036	0.019	-0.001	0.072	0.000	0.000	1.000	0.0550	0.555
L_rostralmiddlefrontal	2886	0.004	0.020	-0.036	0.043	0.001	9.134	1.101	0.8460	0.981
L_superiorfrontal	2879	-0.003	0.019	-0.039	0.033	0.000	0.000	1.000	0.8720	0.981
L_superiorparietal	2851	-0.019	0.020	-0.058	0.019	0.001	4.898	1.052	0.3200	0.910
L_superiortemporal	2733	0.001	0.027	-0.053	0.054	0.008	42.226	1.731	0.9790	0.981
L_supramarginal	2775	-0.022	0.021	-0.063	0.020	0.001	12.048	1.137	0.3060	0.910
L_frontalpole	2902	0.046	0.020	0.007	0.084	0.001	7.506	1.081	0.0210	0.382
L_temporalpole	2864	0.019	0.019	-0.018	0.055	0.000	0.000	1.000	0.3140	0.910
L_transversetemporal	2903	0.035	0.018	-0.001	0.071	0.000	0.000	1.000	0.0600	0.555
L_insula	2829	-0.002	0.022	-0.045	0.041	0.002	18.872	1.233	0.9280	0.981
R_bankssts	2840	0.034	0.031	-0.027	0.096	0.014	58.338	2.400	0.2740	0.910
R_caudalanteriorcingulate	2897	0.025	0.026	-0.026	0.077	0.007	40.506	1.681	0.3320	0.919
R_caudalmiddlefrontal	2884	0.003	0.019	-0.033	0.039	0.000	0.000	1.000	0.8690	0.981
R_cuneus	2793	0.004	0.019	-0.033	0.041	0.000	0.000	1.000	0.8420	0.981
R_entorhinal	2695	0.007	0.020	-0.032	0.046	0.001	4.544	1.048	0.7350	0.981
R_fusiform	2877	-0.001	0.024	-0.047	0.045	0.004	27.295	1.375	0.9730	0.981
R_inferiorparietal	2828	0.024	0.028	-0.031	0.079	0.009	46.709	1.876	0.4000	0.981
R_inferiortemporal	2855	-0.026	0.025	-0.076	0.023	0.006	35.851	1.559	0.2980	0.910

Region	N	Effect Size (r)	SE	Lower CI	Upper CI	tau^2	I^2	H^2	pvalue	FDRp
R_isthmuscingulate	2890	-0.039	0.026	-0.091	0.013	0.007	41.837	1.719	0.1370	0.844
R_lateraloccipital	2868	0.009	0.030	-0.050	0.069	0.012	55.610	2.253	0.7570	0.981
R_lateralorbitofrontal	2853	0.005	0.031	-0.055	0.065	0.013	55.888	2.267	0.8810	0.981
R_lingual	2843	-0.019	0.028	-0.075	0.036	0.009	48.214	1.931	0.4960	0.981
R_medialorbitofrontal	2847	0.067	0.019	0.031	0.103	0.000	0.000	1.000	0.0001	0.017
R_middletemporal	2832	0.031	0.031	-0.030	0.092	0.013	57.434	2.349	0.3170	0.910
R_parahippocampal	2897	-0.003	0.023	-0.048	0.042	0.003	24.683	1.328	0.9100	0.981
R_paracentral	2894	-0.020	0.027	-0.072	0.032	0.007	42.450	1.738	0.4490	0.981
R_parsopercularis	2859	0.006	0.019	-0.031	0.042	0.000	0.000	1.000	0.7670	0.981
R_parsorbitalis	2882	-0.013	0.029	-0.070	0.044	0.010	51.293	2.053	0.6580	0.981
R_parstriangularis	2853	0.008	0.019	-0.029	0.044	0.000	0.000	1.000	0.6810	0.981
R_pericalcarine	2795	0.019	0.028	-0.035	0.074	0.008	45.088	1.821	0.4870	0.981
R_postcentral	2852	-0.017	0.019	-0.053	0.020	0.000	0.000	1.000	0.3760	0.962
R_posteriorcingulate	2900	0.013	0.027	-0.040	0.065	0.007	43.111	1.758	0.6360	0.981
R_precentral	2859	-0.038	0.027	-0.092	0.016	0.008	45.034	1.819	0.1690	0.910
R_precuneus	2886	-0.020	0.030	-0.079	0.039	0.012	54.430	2.194	0.5040	0.981
R_rostralanteriorcingulate	2853	0.025	0.021	-0.017	0.067	0.002	15.669	1.186	0.2420	0.910
R_rostralmiddlefrontal	2867	-0.008	0.024	-0.054	0.039	0.004	27.734	1.384	0.7460	0.981
R_superiorfrontal	2881	0.013	0.021	-0.029	0.055	0.002	15.955	1.190	0.5550	0.981
R_superiorparietal	2866	-0.016	0.028	-0.071	0.040	0.009	48.525	1.943	0.5780	0.981
R_superiortemporal	2802	0.024	0.021	-0.017	0.065	0.001	11.363	1.128	0.2560	0.910
R_supramarginal	2794	0.003	0.025	-0.046	0.051	0.005	32.058	1.472	0.9150	0.981
R_frontalpole	2894	0.050	0.018	0.014	0.086	0.000	0.000	1.000	0.0070	0.145
R_temporalpole	2820	0.022	0.022	-0.021	0.065	0.002	17.066	1.206	0.3100	0.910
R_transversetemporal	2905	0.012	0.024	-0.034	0.058	0.004	27.716	1.383	0.6070	0.981
R_insula	2797	-0.013	0.023	-0.058	0.031	0.003	20.723	1.261	0.5540	0.981
LThickness	2907	-0.016	0.019	-0.053	0.020	0.000	0.000	1.000	0.3830	0.962
RThickness	2907	0.010	0.019	-0.026	0.046	0.000	0.000	1.000	0.5890	0.981

Table S18: Cortical Surface Area Continuous Model

Region	N	Effect Size (r)	SE	Lower CI	Upper CI	tau^2	I^2	H^2	pvalue	FDRp
L_bankssts	2707	0.009	0.021	-0.032	0.050	0.001	8.943	1.098	0.672	0.905
L_caudalanteriorcingulate	2845	-0.006	0.021	-0.047	0.035	0.001	12.394	1.141	0.780	0.980

Region	N	Effect Size (r)	SE	Lower CI	Upper CI	tau^2	I^2	H^2	pvalue	FDRp
L_caudalmiddlefrontal	2848	-0.006	0.019	-0.044	0.032	0.000	4.217	1.044	0.761	0.967
L_cuneus	2738	-0.013	0.019	-0.050	0.025	0.000	0.000	1.000	0.509	0.859
L_entorhinal	2716	-0.027	0.022	-0.070	0.016	0.002	15.371	1.182	0.217	0.856
L_fusiform	2831	-0.043	0.021	-0.084	-0.002	0.001	11.497	1.130	0.038	0.827
L_inferiorparietal	2790	-0.013	0.022	-0.056	0.030	0.002	17.472	1.212	0.552	0.882
L_inferiortemporal	2804	-0.002	0.024	-0.049	0.044	0.004	27.412	1.378	0.922	0.981
L_isthmuscingulate	2859	0.012	0.019	-0.025	0.048	0.000	0.000	1.000	0.529	0.879
L_lateraloccipital	2827	-0.027	0.021	-0.067	0.014	0.001	10.670	1.119	0.195	0.856
L_lateralorbitofrontal	2834	0.007	0.019	-0.030	0.043	0.000	0.000	1.000	0.716	0.932
L_lingual	2803	0.001	0.025	-0.048	0.050	0.005	33.945	1.514	0.964	0.981
L_medialorbitofrontal	2823	-0.037	0.034	-0.103	0.029	0.018	64.449	2.813	0.274	0.856
L_middletemporal	2717	0.027	0.019	-0.010	0.065	0.000	0.911	1.009	0.156	0.856
L_parahippocampal	2848	-0.056	0.020	-0.096	-0.016	0.001	9.139	1.101	0.006	0.205
L_paracentral	2858	0.019	0.028	-0.036	0.075	0.009	48.056	1.925	0.494	0.856
L_parsopercularis	2836	0.013	0.019	-0.024	0.050	0.000	0.000	1.000	0.486	0.856
L_parsorbitalis	2846	0.012	0.023	-0.033	0.057	0.003	23.378	1.305	0.605	0.905
L_parstriangularis	2834	-0.022	0.022	-0.066	0.022	0.003	19.970	1.250	0.322	0.856
L_pericalcarine	2772	-0.015	0.021	-0.055	0.026	0.001	9.724	1.108	0.482	0.856
L_postcentral	2800	0.039	0.025	-0.010	0.087	0.005	32.277	1.477	0.120	0.856
L_posteriorcingulate	2855	0.026	0.019	-0.011	0.063	0.000	0.000	1.000	0.165	0.856
L_precentral	2816	-0.009	0.022	-0.053	0.035	0.003	19.958	1.249	0.675	0.905
L_precuneus	2848	0.002	0.035	-0.066	0.071	0.020	67.255	3.054	0.948	0.981
L_rostralanteriorcingulate	2836	-0.021	0.023	-0.066	0.024	0.003	23.790	1.312	0.358	0.856
L_rostralmiddlefrontal	2847	0.021	0.019	-0.016	0.058	0.000	0.000	1.000	0.260	0.856
L_superiorfrontal	2840	0.005	0.037	-0.067	0.077	0.024	70.694	3.412	0.898	0.981
L_superiorparietal	2812	0.018	0.026	-0.032	0.068	0.006	35.764	1.557	0.478	0.856
L_superiortemporal	2695	0.006	0.023	-0.040	0.051	0.003	21.758	1.278	0.805	0.981
L_supramarginal	2737	0.048	0.032	-0.014	0.110	0.014	57.169	2.335	0.129	0.856
L_frontalpole	2863	-0.011	0.022	-0.053	0.032	0.002	17.213	1.208	0.625	0.905
L_temporalpole	2824	-0.033	0.025	-0.081	0.015	0.005	31.502	1.460	0.180	0.856
L_transversetemporal	2864	-0.018	0.020	-0.057	0.021	0.001	8.165	1.089	0.367	0.856
L_insula	2790	-0.013	0.019	-0.050	0.024	0.000	0.000	1.000	0.476	0.856
R_bankssts	2801	0.019	0.027	-0.034	0.071	0.007	41.195	1.701	0.489	0.856
R_caudalanteriorcingulate	2857	-0.018	0.022	-0.061	0.026	0.002	19.412	1.241	0.420	0.856

Region	N	Effect Size (r)	SE	Lower CI	Upper CI	tau^2	I^2	H^2	pvalue	FDRp
R_caudalmiddlefrontal	2845	-0.026	0.019	-0.062	0.011	0.000	0.000	1.000	0.169	0.856
R_cuneus	2754	-0.015	0.019	-0.052	0.022	0.000	0.000	1.000	0.419	0.856
R_entorhinal	2654	-0.036	0.025	-0.085	0.013	0.005	29.647	1.421	0.152	0.856
R_fusiform	2838	0.003	0.022	-0.041	0.046	0.002	18.627	1.229	0.900	0.981
R_inferiorparietal	2791	0.010	0.021	-0.032	0.052	0.002	14.208	1.166	0.648	0.905
R_inferiortemporal	2816	-0.002	0.025	-0.051	0.048	0.006	35.360	1.547	0.952	0.981
R_isthmuscingulate	2850	0.027	0.024	-0.021	0.075	0.004	30.620	1.441	0.268	0.856
R_lateraloccipital	2829	-0.003	0.029	-0.059	0.053	0.010	49.222	1.969	0.921	0.981
R_lateralorbitofrontal	2814	-0.009	0.023	-0.055	0.036	0.003	24.657	1.327	0.687	0.905
R_lingual	2804	-0.008	0.019	-0.045	0.029	0.000	0.000	1.000	0.678	0.905
R_medialorbitofrontal	2808	-0.062	0.032	-0.124	0.000	0.014	58.977	2.438	0.051	0.856
R_middletemporal	2795	0.000	0.019	-0.037	0.036	0.000	0.000	1.000	0.981	0.981
R_parahippocampal	2858	-0.001	0.022	-0.044	0.041	0.002	17.058	1.206	0.952	0.981
R_paracentral	2855	-0.005	0.024	-0.052	0.043	0.004	30.618	1.441	0.842	0.981
R_parsopercularis	2820	0.017	0.019	-0.020	0.053	0.000	0.000	1.000	0.376	0.856
R_parsorbitalis	2843	-0.028	0.028	-0.083	0.027	0.009	47.357	1.900	0.321	0.856
R_parstriangularis	2814	-0.002	0.019	-0.039	0.035	0.000	0.000	1.000	0.918	0.981
R_pericalcarine	2756	-0.033	0.019	-0.071	0.004	0.000	0.000	1.000	0.078	0.856
R_postcentral	2814	0.035	0.022	-0.009	0.078	0.002	19.102	1.236	0.120	0.856
R_posteriorcingulate	2859	-0.001	0.025	-0.049	0.048	0.005	32.457	1.481	0.976	0.981
R_precentral	2820	-0.001	0.019	-0.037	0.036	0.000	0.000	1.000	0.977	0.981
R_precuneus	2847	0.040	0.032	-0.023	0.104	0.016	61.102	2.571	0.214	0.856
R_rostralanteriorcingulate	2814	-0.009	0.019	-0.046	0.027	0.000	0.000	1.000	0.616	0.905
R_rostralmiddlefrontal	2828	0.023	0.021	-0.018	0.064	0.001	11.952	1.136	0.278	0.856
R_superiorfrontal	2842	-0.005	0.022	-0.047	0.038	0.002	16.903	1.203	0.828	0.981
R_superiorparietal	2827	0.025	0.024	-0.023	0.072	0.005	30.749	1.444	0.317	0.856
R_superiortemporal	2763	0.029	0.030	-0.030	0.087	0.011	52.633	2.111	0.338	0.856
R_supramarginal	2756	-0.009	0.029	-0.066	0.048	0.010	49.241	1.970	0.749	0.963
R_frontalpole	2854	0.021	0.021	-0.020	0.063	0.002	14.926	1.175	0.314	0.856
R_temporalpole	2781	-0.014	0.019	-0.051	0.023	0.000	0.000	1.000	0.465	0.856
R_transversetemporal	2866	-0.011	0.019	-0.047	0.025	0.000	0.000	1.000	0.551	0.882
R_insula	2758	-0.003	0.021	-0.043	0.038	0.001	9.129	1.100	0.892	0.981
LSurfArea	2868	-0.014	0.020	-0.052	0.025	0.001	5.627	1.060	0.487	0.856
RSurfArea	2868	0.014	0.020	-0.025	0.052	0.001	5.627	1.060	0.487	0.856

Table S19: Subcortical Correlation Model

Region	N	Effect Size (r)	SE	Lower CI	Upper CI	tau^2	I^2	H^2	pvalue	FDRp
LLatVent	2990	0.002	0.023	-0.043	0.046	0.003	24.698	1.328	0.939	0.985
RLatVent	2990	0.001	0.019	-0.036	0.039	0.001	6.043	1.064	0.944	0.985
Lthal	2966	0.007	0.030	-0.051	0.066	0.012	55.391	2.242	0.806	0.967
Lcaud	2981	0.017	0.024	-0.029	0.064	0.004	29.879	1.426	0.460	0.923
Lput	2934	-0.012	0.018	-0.048	0.024	0.000	0.000	1.000	0.522	0.923
Lpal	2762	-0.023	0.019	-0.060	0.014	0.000	0.000	1.000	0.226	0.923
Lhippo	2970	0.014	0.018	-0.022	0.050	0.000	0.000	1.000	0.443	0.923
Lamyg	2972	0.010	0.018	-0.026	0.045	0.000	0.000	1.000	0.601	0.923
Laccumb	2965	0.012	0.020	-0.027	0.052	0.001	10.789	1.121	0.547	0.923
Rthal	2986	0.014	0.031	-0.047	0.075	0.014	59.948	2.497	0.654	0.923
Rcaud	2982	0.013	0.027	-0.039	0.065	0.007	43.636	1.774	0.625	0.923
Rput	2965	0.000	0.018	-0.036	0.036	0.000	0.000	1.000	0.985	0.985
Rpal	2970	-0.032	0.018	-0.067	0.004	0.000	0.000	1.000	0.084	0.923
Rhippo	2978	0.022	0.018	-0.014	0.058	0.000	0.000	1.000	0.229	0.923
Ramyg	2960	0.019	0.024	-0.029	0.067	0.005	33.861	1.512	0.444	0.923
Raccumb	2963	0.020	0.023	-0.025	0.064	0.003	24.514	1.325	0.388	0.923

Funding and Acknowledgments

MK acknowledges support from the National Bank Fellowship (McGill University) and the Swiss National Foundation (P2SKP3_178175). GM was supported by a Sir Henry Dale Fellowship jointly funded by the Wellcome Trust and the Royal Society (grant number 202397/Z/16/Z). MG was supported by NHMRC as an R.D. Wright Biomedical Career Development Fellow (#1061875). CP was supported by NHMRC Senior Principal Research Fellowships (#628386 & #1105825) and a NHMRC Program Grant (ID: 1150083). AF was supported by the Sylvia and Charles Viertel Charitable Foundation; and the National Health and Medical Research Council (ID: 1050504). MAB is supported by a Senior Research Fellowship from the Australian National Health and Medical Research Council (NHMRC). AK was supported by the German Research Foundation (KR 3822/5-1, KR 3822/7-2). YW was supported by the National Natural Science Foundation of China (31871114), CAS Key Laboratory of Mental Health, Institute of Psychology. ET was supported by the German Research Foundation (DFG 31/2-1). TK was funded by the German Research Foundation (DFG FOR2107 KI588/14-1 and KI588/14-2). IL was funded by RBRF (20-013-00748). SL acknowledges funding from Fonds de la Recherche du Québec – Santé (FRQ-S) and the Canadian Institutes of Health Research (CIHR). PK was funded by a University of Roehampton Vice Chancellor Scholarship. SK was funded by the Swiss National Science Foundation. IN was funded by the DFG: FOR2107, FSU Jena: Junior Scientist Programme, UKGM: FoFoe. MD was funded by the Schweizerischer Nationalfonds zur Förderung der Wissenschaftlichen Forschung (Grant/Award Number: 100019_159440). PD was supported by the National Institute of Mental Health (NIMH R00 MH086756; NIMH P50 MH080173). WR was funded by Collegium Helveticum, Transdisciplinary Research Institute of ETH Zurich and University Zurich. MJG was supported by National Health and Medical Research Council (NHMRC) Project Grants 630471 and 1081603. AD was supported by the Canadian Institutes of Health Research. JT was supported by the NIMH (5R01MH116147 (PI P. Thompson). BB was funded by the Canada Research Chairs Program.

FOR2107: This work was funded by the German Research Foundation (DFG, grant FOR2107 DA1151/5-1 and DA1151/5-2 to UD; SFB-TRR58, Projects C09 and Z02 to UD) and the Interdisciplinary Center for Clinical Research (IZKF) of the medical faculty of Münster (grant Dan3/012/17 to UD).

DECOP: This study was funded by the Netherland Organization for Scientific Research (NWO) [Veni #451-13-035].

ASRB: The Australian Schizophrenia Research Bank (ASRB) was supported by the National Health and Medical Research Council of Australia (NHMRC) (Enabling Grant, ID 386500), the Pratt Foundation, Ramsay Health Care, the Viertel Charitable Foundation and the Schizophrenia Research Institute. Chief Investigators for ASRB were Carr, V., Schall, U., Scott, R., Jablensky, A., Mowry, B., Michie, P., Catts, S., Henskens, F., Pantelis, C. We thank Loughland, C., the ASRB Manager, and acknowledge the help of Jason Bridge for ASRB database queries.

IGP: This study was funded by Project Grants from the Australian National Health and Medical Research Council of Australia (NHMRC; APP630471 and APP1081603), the Macquarie University's Australian Research Council Centre of Excellence in Cognition and its Disorders (CE110001021).

London1a: This work was supported by a Brain & Behavior Research Foundation NARSAD Young Investigator Grant to GM (#21200, Lieber Investigator).

London1b: Funding for this study was provided by European Science Foundation EURYI grant (NWO 044035001 to AA).

AUCK: This study was funded by the Faculty Research Development Fund (No. 3702215) and the MRI Pilot Study Fund (Skyra 25- 001-A) from the University of Auckland.

CAM: This work was supported by the Wellcome Trust (to PM and WS) and the Niels Stensen Foundation (to KMJD).
